# Supplementary material for: Effectiveness of electrophysical modalities in the sensorimotor rehabilitation of radial, ulnar, and median neuropathies: A meta-analysis
Source: PLoS One. 2021 Mar 18;16(3):e0248484. doi: 10.1371/journal.pone.0248484 (PMC7971482; doi:10.1371/journal.pone.0248484)
Supplement: S2 Table — (DOCX) [file pone.0248484.s002.docx]

**PICO for the effectiveness of electrophysical therapies in the treatment of ulnar, radial, or median neuropathies.**

| **Population:** | People with radial, median, or ulnar neuropathy |
| --- | --- |
| **Intervention:** | Provision of treatments with electrotherapy modalities (Low-level laser therapy, ultrasound, magnetotherapy, radial extracorporeal shockwave, and others.) |
| **Control:** | Provision of treatment with placebo, physical or electrophysical therapy. |
| **Outcomes (primary):** | Pain, symptom severity, functional status, neurophysiological parameters (distal motor latency, the amplitude of motor action potential, motor conduction velocity, distal sensory latency, the amplitude of sensory action potential, and sensory conduction velocity) |
| **Outcomes (secondary):** | Grip and pinch strength. |
