# Supplementary material for: Effectiveness of electrophysical modalities in the sensorimotor rehabilitation of radial, ulnar, and median neuropathies: A meta-analysis
Source: PLoS One. 2021 Mar 18;16(3):e0248484. doi: 10.1371/journal.pone.0248484 (PMC7971482; doi:10.1371/journal.pone.0248484)
Supplement: S3 Table — (PDF) [file pone.0248484.s003.pdf]

Electrophysical compared to placebo

Author(s): Bula Oyola, Ena Lucía; Belda Lois, Juan Manuel; Porcar Seder, Rosa; Page Del Pozo, Álvaro
Question: Electrophysical therapy modalities compared to placebo for radial, ulnar or median neuropathies
Setting:
Bibliography:

Certainty assessment							№ of patients		Effect		Certainty	Importance
№ of studies	Study design	Risk of bias	Inconsistency	Indirectness	Imprecision	Other considerations	electrophysical therapy modalities	placebo	Relative (95% CI)	Absolute (95% CI)		
**Pain (VAS) (follow up: range 2 weeks to 18; assessed with: Visual analog scale; Scale from: 0 to 10)**												
12	randomised trials	serious [a]	serious [b]	not serious	not serious	publication bias strongly suspected [c]	352	348	-	SMD **0.89 SD lower** (1.79 lower to 0.02 higher)	⊕◯◯◯ VERY LOW	IMPORTANT
**Symptom Severity (follow up: range 2 weeks to 18 weeks; assessed with: Symptom Severity Scale; Scale from: 1 to 5)**												
11	randomised trials	serious [a]	serious [b]	not serious	not serious	publication bias strongly suspected [c]	374	373	-	SMD **1.01 SD lower** (1.65 lower to 0.37 lower)	⊕◯◯◯ VERY LOW	IMPORTANT
**Functional Status (follow up: range 2 weeks to 18 weeks; assessed with: Functional Status Scale; Scale from: 1 to 5)**												
10	randomised trials	serious [a]	serious [b]	not serious	not serious	publication bias strongly suspected [c]	320	319	-	SMD **0.79 SD lower** (1.45 lower to 0.13 lower)	⊕◯◯◯ VERY LOW	IMPORTANT
**Motor Latency (follow up: range 2 weeks to 18 weeks)**												
14	randomised trials	serious [d]	serious [b]	not serious	not serious	publication bias strongly suspected [c]	458	454	-	SMD **0.31 SD lower** (0.66 lower to 0.04 higher)	⊕◯◯◯ VERY LOW	IMPORTANT
**Sensory Latency (follow up: range 2 weeks to 18 weeks)**												
11	randomised trials	serious [d]	serious [b]	not serious	not serious	publication bias strongly suspected [c]	358	355	-	SMD **0.03 SD higher** (0.29 lower to 0.35 higher)	⊕◯◯◯ VERY LOW	IMPORTANT
**Motor Nerve Conduction Velocity (follow up: range 2 weeks to 18 weeks)**												

Certainty assessment							№ of patients		Effect		Certainty	Importance
№ of studies	Study design	Risk of bias	Inconsistency	Indirectness	Imprecision	Other considerations	electrophysical therapy modalities	placebo	Relative (95% CI)	Absolute (95% CI)		
6	randomised trials	serious [d]	serious [b]	not serious	not serious	none	177	177	-	SMD **0.27 SD higher** (0.25 lower to 0.8 higher)	⊕⊕◯◯ LOW	IMPORTANT
**Sensory Nerve Conduction Velocity (follow up: range 2 weeks to 18 weeks)**												
12	randomised trials	serious [a]	serious [b]	not serious	not serious	publication bias strongly suspected [c]	360	359	-	SMD **0.09 SD lower** (0.57 lower to 0.38 higher)	⊕◯◯◯ VERY LOW	IMPORTANT
**Compound Muscle Action Potential Amplitude (follow up: range 2 weeks to 18 weeks)**												
5	randomised trials	serious [d]	serious [b]	not serious	not serious	none	186	185	-	SMD **0.15 SD higher** (0.41 lower to 0.72 higher)	⊕⊕◯◯ LOW	IMPORTANT
**Sensory Nerve Action Potential Amplitude (follow up: range 2 weeks to 18 weeks)**												
4	randomised trials	serious [d]	serious [b]	not serious	not serious	none	167	166	-	SMD **0.28 SD higher** (0.06 lower to 0.62 higher)	⊕⊕◯◯ LOW	IMPORTANT
**Grip Strength (follow up: range 2 weeks to 18 weeks; assessed with: Dynamometry)**												
6	randomised trials	serious [a]	serious [b]	not serious	not serious	none	228	226	-	SMD **0.08 SD higher** (0.22 lower to 0.37 higher)	⊕⊕◯◯ LOW	IMPORTANT
**Pinch Strength (follow up: range 2 weeks to 18 weeks; assessed with: Dynamometry)**												
3	randomised trials	serious [a]	serious [b]	not serious	not serious	none	114	113	-	SMD **0.57 SD higher** (0.26 lower to 1.41 higher)	⊕⊕◯◯ LOW	IMPORTANT

CI: Confidence interval; **SMD:** Standardised mean difference

Explanations

a. Risk of bias in blinding of participants, personnel and assessors and incomplete information about dropouts.

b. Heterogeneity >50%.

c. Risk of publication bias identified by funnel plot.

d. Risk of bias in random sequence generation, allocation concealment, and blinding of participants, personnel and assessors.

Electrophysical compared to manual therapy

Author(s): Bula Oyola, Ena Lucía; Belda Lois, Juan Manuel; Porcar Seder, Rosa; Page Del Pozo, Álvaro
Question: Electrophysical therapy modalities compared to manual therapy for radial, ulnar or median neuropathies
Setting:
Bibliography:

Certainty assessment							№ of patients		Effect		Certainty	Importance
№ of studies	Study design	Risk of bias	Inconsistency	Indirectness	Imprecision	Other considerations	electrophysical therapy modalities	manual therapy	Relative (95% CI)	Absolute (95% CI)		
**Pain (VAS) (follow up: range 2 weeks to 18 weeks; assessed with: Visual analog scale; Scale from: 0 to 10)**												
3	randomised trials	serious [a]	serious [b]	not serious	not serious	none	62	62	-	SMD **0.19 SD higher** (2.39 lower to 2.77 higher)	⨁⨁◯◯ LOW	IMPORTANT
**Symptom Severity (follow up: range 2 weeks to 18 weeks; assessed with: Symptom Severity Scale; Scale from: 1 to 5)**												
3	randomised trials	serious [a]	serious [b]	not serious	not serious	none	117	117	-	SMD **1.44 SD higher** (0.27 lower to 3.15 higher)	⨁⨁◯◯ LOW	IMPORTANT
**Functional Status (follow up: range 2 weeks to 18 weeks; assessed with: Functional Status Scale; Scale from: 1 to 5)**												
3	randomised trials	serious [a]	serious [b]	not serious	not serious	none	117	117	-	SMD **0.99 SD higher** (0.1 higher to 1.89 higher)	⨁⨁◯◯ LOW	IMPORTANT
**Motor Latency (follow up: range 2 weeks to 18 weeks)**												
3	randomised trials	serious [c]	serious [b]	not serious	not serious	none	97	97	-	SMD **0.47 SD lower** (1.51 lower to 0.56 higher)	⨁⨁◯◯ LOW	IMPORTANT
**Sensory Latency (follow up: range 2 weeks to 18 weeks)**												
2	randomised trials	serious [c]	serious [b]	not serious	not serious	none	27	27	-	SMD **0.48 SD lower** (1.74 lower to 0.78 higher)	⨁⨁◯◯ LOW	IMPORTANT
**Sensory Nerve Conduction Velocity (follow up: range 2 weeks to 18 weeks)**												

Certainty assessment							№ of patients		Effect		Certainty	Importance
№ of studies	Study design	Risk of bias	Inconsistency	Indirectness	Imprecision	Other considerations	electrophysical therapy modalities	manual therapy	Relative (95% CI)	Absolute (95% CI)		
2	randomised trials	serious [d]	serious [b]	not serious	not serious	none	85	85	-	SMD **0.61 SD higher** (0.07 lower to 1.3 higher)	⨁⨁◯◯ LOW	IMPORTANT
**Grip Strength (follow up: range 2 weeks to 18 weeks; assessed with: Dynamometry)**												
2	randomised trials	serious [d]	serious [b]	not serious	not serious	none	27	27	-	SMD **0.89 SD lower** (2.49 lower to 0.71 higher)	⨁⨁◯◯ LOW	IMPORTANT

CI: Confidence interval; **SMD:** Standardised mean difference

Explanations

a. Risk of bias in random sequence generation, allocation concealment, incomplete information on drop-outs, and blinding of participants, personnel and assessors.
b. Heterogeneity >50%.
c. Risk of bias in allocation concealment and blinding of participants, personnel and assessors.
d. Risk of bias in blinding of participants, personnel and assessors and incomplete information on drop-outs.

Electrophysical compared to splinting

Author(s): Bula Oyola, Ena Lucía; Belda Lois, Juan Manuel; Porcar Seder, Rosa; Page Del Pozo, Álvaro
Question: Electrophysical therapy modalities compared to splinting for radial, ulnar or median neuropathies
Setting:
Bibliography:

Certainty assessment							№ of patients		Effect		Certainty	Importance
№ of studies	Study design	Risk of bias	Inconsistency	Indirectness	Imprecision	Other considerations	electrophysical therapy modalities	splinting	Relative (95% CI)	Absolute (95% CI)		
**Pain (VAS) (follow up: range 2 weeks to 18 weeks; assessed with: Visual analog scale; Scale from: 0 to 10)**												
4	randomised trials	serious [a,b]	serious [c]	not serious	not serious	none	83	78	-	SMD **0.77 SD lower** (1.59 lower to 0.05 higher)	⨁⨁◯◯ LOW	IMPORTANT
**Symptom Severity (follow up: range 2 weeks to 18 weeks; assessed with: Symptom Severity Scale; Scale from: 1 to 5)**												
3	randomised trials	serious [a,b]	serious [c]	not serious	not serious	none	57	55	-	SMD **0.66 SD lower** (1.33 lower to 0.01 higher)	⨁⨁◯◯ LOW	IMPORTANT
**Functional Status (follow up: range 2 weeks to 18 weeks; assessed with: Functional Status Scale; Scale from: 1 to 5)**												
3	randomised trials	serious [b,d]	serious [c]	not serious	not serious	none	57	55	-	SMD **0.55 SD lower** (1.2 lower to 0.11 higher)	⨁⨁◯◯ LOW	IMPORTANT
**Motor Latency (follow up: range 2 weeks to 18 weeks)**												
2	randomised trials	serious [b,d]	serious [c]	not serious	not serious	none	39	41	-	SMD **0.31 SD lower** (1.5 lower to 0.88 higher)	⨁⨁◯◯ LOW	IMPORTANT
**Sensory Nerve Conduction Velocity (follow up: range 2 weeks to 18 weeks)**												
3	randomised trials	serious [b,d]	serious [c]	not serious	not serious	none	57	59	-	SMD **0.53 SD higher** (0.42 lower to 1.48 higher)	⨁⨁◯◯ LOW	IMPORTANT

CI: Confidence interval; **SMD:** Standardised mean difference

Explanations

a. Risk of bias in random sequence generation, incomplete information on drop-outs, incomplete outcome data (intention to include in analysis) and selective reporting.
b. Since the comparator was a splint, concealment and blinding were not feasible. These risks were ruled out.
c. Heterogeneity >50%.
d. Risk of bias by incomplete information on drop-outs, incomplete outcome data (intention to include in analysis) and selective reporting.

Low-level laser therapy compared to other electrophysical modalities

Author(s): Bula Oyola, Ena Lucía; Belda Lois, Juan Manuel; Porcar Seder, Rosa; Page Del Pozo, Álvaro
Question: Low-level laser therapy compared to other electrophysical modalities for radial, ulnar or median neuropathies
Setting:
Bibliography:

Certainty assessment							№ of patients		Effect		Certainty	Importance
№ of studies	Study design	Risk of bias	Inconsistency	Indirectness	Imprecision	Other considerations	low-level laser therapy	other electrophysical modalities	Relative (95% CI)	Absolute (95% CI)		
**Pain (VAS) (follow up: range 2 weeks to 18 weeks; assessed with: Visual analog scale; Scale from: 0 to 10)**												
3	randomised trials	serious [a]	serious [b]	not serious	not serious	none	85	85	-	SMD **1.11 SD higher** (0.52 lower to 2.75 higher)	⨁⨁◯◯ LOW	IMPORTANT
**Motor Latency (follow up: range 2 weeks to 18 weeks)**												
4	randomised trials	serious [c]	serious [b]	not serious	not serious	none	103	100	-	SMD **1.42 SD higher** (1.3 lower to 4.14 higher)	⨁⨁◯◯ LOW	IMPORTANT
**Sensory Nerve Conduction Velocity (follow up: range 2 weeks to 18 weeks)**												
2	randomised trials	serious [c]	not serious	not serious	not serious	none	28	25	-	SMD **0.56 SD higher** (0.01 higher to 1.12 higher)	⨁⨁⨁◯ MODERATE	IMPORTANT

CI: Confidence interval; **SMD:** Standardised mean difference

Explanations

a. Risk of bias in allocation concealment, selective reporting, and blinding of participants and personnel.
b. Heterogeneity >50%.
c. Risk of bias in random sequence generation, allocation concealment, incomplete information on drop-outs, selective reporting, and blinding of participants and personnel.

Ultrasound therapy compared to other electrophysical modalities

Author(s): Bula Oyola, Ena Lucía; Belda Lois, Juan Manuel; Porcar Seder, Rosa; Page Del Pozo, Álvaro
Question: Ultrasound therapy compared to other electrophysical modalities for radial, ulnar or median neuropathies
Setting:
Bibliography:

Certainty assessment							№ of patients		Effect		Certainty	Importance
№ of studies	Study design	Risk of bias	Inconsistency	Indirectness	Imprecision	Other considerations	ultrasound therapy	other electrophysical modalities	Relative (95% CI)	Absolute (95% CI)		
**Symptom Severity (follow up: range 2 weeks to 18 weeks; assessed with: Symptom Severity Scale; Scale from: 1 to 5)**												
3	randomised trials	serious [a]	serious [b]	not serious	not serious	none	82	84	-	SMD **0.11 SD lower** (1.05 lower to 0.83 higher)	⨁⨁◯◯ LOW	IMPORTANT
**Functional Status (follow up: range 2 weeks to 18 weeks; assessed with: Functional Status Scale; Scale from: 1 to 5)**												
3	randomised trials	serious [a]	not serious [b]	not serious	not serious	none	82	84	-	SMD **0.04 SD lower** (0.34 lower to 0.27 higher)	⨁⨁⨁◯ MODERATE	IMPORTANT

CI: Confidence interval; **SMD:** Standardised mean difference

Explanations

a. Risk of bias in random sequence generation, allocation concealment, incomplete information on drop-outs, selective reporting, and blinding of participants and personnel.
b. Heterogeneity >50%.

Low-level laser therapy compared to Ultrasound therapy

Author(s): Bula Oyola, Ena Lucía; Belda Lois, Juan Manuel; Porcar Seder, Rosa; Page Del Pozo, Álvaro
Question: Low-level laser therapy compared to ultrasound therapy for radial, ulnar or median neuropathy
Setting:
Bibliography:

Certainty assessment							№ of patients		Effect		Certainty	Importance
№ of studies	Study design	Risk of bias	Inconsistency	Indirectness	Imprecision	Other considerations	low-level laser therapy	ultrasound therapy	Relative (95% CI)	Absolute (95% CI)		
**Sensory Latency (follow up: range 2 weeks to 18 weeks)**												
2	randomised trials	serious [a]	serious [b]	not serious	not serious	none	75	75	-	SMD **1.65 SD lower** (3.66 lower to 0.36 higher)	⨁⨁◯◯ LOW	IMPORTANT
**Grip Strength (follow up: range 2 weeks to 18 weeks; assessed with: Dynamometry)**												
2	randomised trials	serious [c]	serious [b]	not serious	not serious	none	39	40	-	SMD **1.25 SD lower** (2.23 lower to 0.27 lower)	⨁⨁◯◯ LOW	IMPORTANT

CI: Confidence interval; **SMD:** Standardised mean difference

Explanations

a. Risk of bias in blinding of participants and personnel, and selective reporting.
b. Heterogeneity >50%.
c. Risk of bias in allocation concealment, blinding of participants and personnel, and incomplete information on drop-outs.
