# Supplementary material for: Effectiveness of electrophysical modalities in the sensorimotor rehabilitation of radial, ulnar, and median neuropathies: A meta-analysis
Source: PLoS One. 2021 Mar 18;16(3):e0248484. doi: 10.1371/journal.pone.0248484 (PMC7971482; doi:10.1371/journal.pone.0248484)
Supplement: S5 Table — (DOCX) [file pone.0248484.s005.docx]

# S5 Table. Measures and outcomes of included studies.

Abbreviations:

**VAS:** Visual Analog Scale

**FSS:** Functional Status Scale

**SSS**: Symptom Severity Scale

**DML**: Distal Motor Latency

**ML***:* Motor Latency

**SDL***:* Sensory Distal Latency

**SPL**: Sensory Peak Latency

**SL**: Sensory Latency

**MNCV**: Motor Nerve Conduction Velocity

**SNCV**: Sensory Nerve Conduction Velocity

**CMAP**: Compound Muscle Action Potential

**SNAP**: Sensory Nerve Action Potential

**TMT**: Treatment

**WK**: Week

**MO**: Month

**LLLT**: Low-level laser therapy

**US:** Ultrasound

**ESWT**: Extracorporeal shock wave therapy

**SMF**: Static Magnetic Field

**PMF**: Pulsed Magnetic Field

**TENS**: Transcutaneous Electrical Nerve Stimulation

**IFC**: Interferential Current Therapy

**PRF**: Pulsed Radiofrequency

| **S5 Table. Measures and outcomes of included studies.** | | | | | | | | | | |
| --- | --- | --- | --- | --- | --- | --- | --- | --- | --- | --- |
| Author | Study Design | Lesion and severity | Gender F/M | Intervention | Comparator/control | SMD | Statistical method | Outcome measures and follow-up | P-value | Outcomes |
| **Jiang et al.** | Randomized placebo-controlled, double-blind study | CTS. Mild to moderate | N/R | Group 1: LLLT. Painless Light PL-830, wavelength 830 nm, frequency 10 Hz, mean power 60 mW (2 × 30 mW), treatment dosage 9.7 J/cm^2^. 10 min sessions, 5 times/wk for 2 weeks; n = 45 | Group 2: Placebo laser;  n = 42 | Mild -1.62 (-2.24; -1.00)  Mod -0.20  (-0.88; 0.49) | Wilcoxon´s test | VAS  (baseline, 2wk, 5wk) | (Mild) The between-group difference at 2wk p<0.001 and p<0.05 at 5wk.  (mod) The between-group difference at 2wk p<0.01. | Mild groups: LLLT group mean change ± SD from baseline to 2wk -2.76 ± 1.48, from 2wk to 5wk -1.01 ± 0.91; placebo group means change ± SD from baseline to 2wk -0.50 ± 0.83, from 2wk to 5wk 0.01 ± 0.71.  Moderate groups: LLLT group mean change ± SD from baseline to 2wk -3.17±1.81, from 2wk to 5wk -1.25 ± 0.78; placebo group means change ± SD from baseline to 2wk -2.01 ± 0.91, from 2wk to 5wk -0.51 ± 0.86. |
|  |  |  |  |  |  | Mild -0.71 (-1.26; -0.15) Mod -0.24  (-0.77; 0.30) |  | SSS  (baseline, 2wk, 5wk) | (Mild) Between-group difference at 2wk p<0.001. | Mild groups: LLLT group mean change ± SD from baseline to 2wk -0.78 ± 0.31, from 2wk to 5wk -0.06 ± 0.53; placebo group means change ± SD from baseline to 2wk - 0.12 ± 0.25, from 2wk to 5wk 0.31 ± 0.49.  Moderate groups: LLLT group mean change ± SD from baseline to 2wk -0.91 ± 0.32, from 2wk to 5wk -0.47 ± 0.41; placebo group means change ± SD from baseline to 2wk -0.29 ± 0.76, from 2wk to 5wk 0.31 ± 0.72. |
|  |  |  |  |  |  | Mild -0.66 (-1.21; -0.11) Mod -1.90  (-2.75; -1.06) |  | DML  (baseline, 2wk, 5wk) | (Mild) Between-group difference at 2wk p<0.01. | Mild groups: LLLT group mean change ± SD from baseline to 2wk -0.29 ± 0.21, from 2wk to 5wk 0.01 ± 0.11; placebo group means change ± SD from baseline to 2wk -0.16 ± 0.14, from 2wk to 5wk -0.03 ± 0.25.  Moderate groups: LLLT group mean change ± SD from baseline to 2wk -0.07 ± 0.04, from 2wk to 5wk -0.07±0.03; placebo group means change ± SD from baseline to 2wk -0.04 ± 0.13, from 2wk to 5wk -0.02 ± 0.06. |
|  |  |  |  |  |  | Mild -0.87 (-1.43; -0.31) Mod 1.28 (0.52; 2.04) |  | SPL  (baseline, 2wk, 5wk) | (Mild) Between-group difference at 2wk p<0.01. | Mild groups: LLLT group mean change ± SD from baseline to 2wk -0.24 ± 0.12, from 2wk to 5wk -0.02 ± 0.23; placebo group means change ± SD from baseline to 2wk -0.08 ± 0.13, from 2wk to 5wk 0.01 ± 0.04.  Moderate groups: LLLT group mean change ± SD from baseline to 2wk -0.08 ± 0.05, from 2wk to 5wk -0.14 ± 0.06; placebo group means change ± SD from baseline to 2wk -0.05 ± 0.04, from 2wk to 5wk -0.04 ± 0.07. |

| **S5 Table.** (continued) | | | | | | | | | | |
| --- | --- | --- | --- | --- | --- | --- | --- | --- | --- | --- |
| Author | Study Design | Lesion and severity | Gender F/M | Intervention | Comparator/control | SMD | Statistical method | Outcome measures and follow-up | P-value | Outcomes |
| **Abid Ali et al.** | Randomized controlled trial | CTS. Mild to moderate | G1 and G2: 33/27  G3: N/R | Group 1: LLLT by Gallium – Arsenide (Ga-As) laser, wavelength 904 nm in pulsed mode, frequency 5000 Hz, maximum output power 15 mW. Sessions, 5 times/wk for 2 weeks;  n = 30 | Group 2: Sham laser;  n = 30  Group 3: Real laser on healthy participants n = 30 | -6.35  (-7.62;  -5.07) | One way  ANOVA | VAS (baseline, 2wk, 4wk) | p< 0.001 | LLLT group mean ± SD from 71 ± 8.9 at baseline to 25.5 ± 6.8 at 2wk to 20.6 ± 4.9 at 4wk. Sham group means ± SD from 58.3 ± 6.9 at baseline to 63.6 ± 4.9 at 2wk to 77 ± 5.3 at 4wk. Control group mean ± SD was 0 at baseline, 2wk, and 4wk. |
|  |  |  |  |  |  | -2.94 (-3.68;  -2.20) |  | SSS (baseline, 2wk, 4wk) | p< 0.001 | LLLT group mean ± SD from 35.9 ± 5.2 at baseline to 23.8 ± 3.7 at 2wk to 21.8 ± 2.8 at 4wk. Sham group means ± SD from 36.6 ± 5 at baseline to 36.9 ± 5 at 2wk to 37.2 ± 4.4 at 4wk. Control group mean ± SD was 11 ± 0 at baseline, 2wk, and 4wk. |
|  |  |  |  |  |  | -1.20 (-1.75;  -0.65) |  | FSS (baseline, 2wk, 4wk) | p< 0.001 | LLLT group mean ± SD from 24.2 ± 8.1 at baseline to 19.7 ± 7.5 at 2wk to 18.8 ± 8.0 at 4wk. Sham group mean ± SD from 27.7 ± 3.6 at baseline to 27.0 ± 4.0 at 2wk to 28.5 ± 3.5 at 4wk. Control group mean ± SD was 8 ± 0 at baseline, 2wk, and 4wk. |
|  |  |  |  |  |  | 0.27 (-0.24; 0.78) |  | DSL (baseline, 2wk, 4wk) | p< 0.001 | LLLT group mean ± SD from 4.2 ± 0.2 at baseline to 4.1 ± 0.15 at 2wk to 3 ± 0.13 at 4wk. Sham group means ± SD from 3.8 ± 0.9 at baseline to 4 ± 0.5 at 2wk to 4.1 ± 0.1 at 4wk. Control group mean ± SD from 1.8 ± 0.25 at baseline to 1.7 ± 0.13 at 2wk to 1.6 ± 0.16 at 4wk. |
| (Continued) | | | | | | | | | | |

| (Continued) | | | | | | | | | | |
| --- | --- | --- | --- | --- | --- | --- | --- | --- | --- | --- |
| **Abid Ali et al.** | Randomized controlled trial | CTS. Mild to moderate | G1 and G2: 33/27  G3: N/R | Group 1: LLLT by Gallium – Arsenide (Ga-As) laser, wavelength 904 nm in pulsed mode, frequency 5000 Hz, maximum output power 15 mW. Sessions, 5 times/wk for 2 weeks;  n = 30 | Group 2: Sham laser; n = 30  Group 3: Real laser on healthy participants; n = 30 | -0.55 (-1.06;  -0.03) | One way  ANOVA | SNCV (baseline, 2wk, 4wk) | p< 0.001 | LLLT group mean ± SD from 39.9 ± 1.2 at baseline to 38.9 ± 2.3 at 2wk to 38.1 ± 1.9 at 4wk. Sham group means ± SD from 40.5 ± 0.9 at baseline to 39.9 ± 1.1 at 2wk to 39.5 ± 0.4 at 4wk. Control group mean ± SD from 57.7 ± 3.9 at baseline to 55.9 ± 2.5 at 2wk to 56.3 ± 3.2 at 4wk. |
|  |  |  |  |  |  | 0.68 (0.16; 1.20) |  | SNAP amplitude (baseline, 2wk, 4wk) | p< 0.001 | LLLT group mean ± SD from 19 ± 4.5 at baseline to 19.3 ± 5.4 at 2wk to 24.5 ± 5.7 at 4wk. Sham group means ± SD from 17 ± 5.5 at baseline to 16.1 ± 3.8 at 2wk to 15.7 ± 4.2 at 4wk. Control group mean ± SD from 39.6 ± 4.3 at baseline to 41.3 ± 5.2 at 2wk to 41.7 ± 6.6 at 4wk. |
|  |  |  |  |  |  | -1.43 (-2.00;  -0.86) |  | DML (baseline, 2wk, 4wk) | p< 0.001 | LLLT group mean ± SD from 5 ± 0.8 at baseline to 4.5 ± 0.4 at 2wk to 4.1 ± 0.1 at 4wk. Sham group means ± SD from 4.9 ± 0.5 at baseline to 5.7 ± 1.1 at 2wk to 5.09 ± 0.9 at 4wk. Control group mean ± SD from 2.5 ± 0.1 at 4wk. |
|  |  |  |  |  |  | 2.10 (1.46; 2.74) |  | MNCV (baseline, 2wk, 4wk) | p< 0.001 | LLLT group mean ± SD from 47 ± 2.5 at baseline to 45 ± 3.2 at 2wk to 44 ± 2.4 at 4wk. Sham group means ± SD from 41 ± 3.3 at baseline to 39.1 ± 2.2 at 2wk to 40.5 ± 2.1 at 4wk. Control group mean ± SD from 58.4 ± 3.2 at baseline to 53 ± 3.2 at 2wk to 55 ± 2.1 at 4wk. |
|  |  |  |  |  |  | 1.77 (1.17; 2.37) |  | CMAP amplitude (baseline, 2wk, 4wk) | p< 0.001 | LLLT group mean ± SD from 8 ± 0.6 at baseline to 9.7 ± 1.1 at 2wk to 10.1 ± 0.6 at 4wk. Sham group means ± SD from 8.3 ± 1.5 at baseline to 7.9 ± 0.9 at 2wk to 8 ± 0.7 at 4wk. Control group mean ± SD from 11.5 ± 2.2 at baseline to 13.8 ± 2.1 at 2wk to 12.9 ± 1.9 at 4wk. |

| **S5 Table.** (continued) | | | | | | | | | | |
| --- | --- | --- | --- | --- | --- | --- | --- | --- | --- | --- |
| Author | Study Design | Lesion and severity | Gender F/M | Intervention | Comparator/  control | SMD | Statistical method | Outcome measures and follow-up | P-value | Outcomes |
| **Tascioglu et al.** | Randomized placebo-controlled, double-blind study | CTS. Not stated | Global:  46/14 | Group 1: LLLT, dosage 90 J. Gal-Al-As diode laser, power output 50 mW, wavelength 830 nm. Dose per tender joint 1.2 J, total dose per treatment 6 J, accumulated dose 90 J. 5 times per week for 3 wk;  n = 20 | Group 2: LLLT, dosage 45 J. Active laser, dose per tender joint was 0.6 J, total dose per treatment 3 J, accumulated dose 45 J total. 5 times per week for 3 wk; n = 20  Group 3: placebo (0 J). 5 times per week for 3 wk; n = 20 | Dosage 90 J  -0.28 (-0.90; 0.34)  Dosage 45 J  -0.49 (-1.12; 0.14) | One way  ANOVA to compare the differences among the three groups with the baseline.  Chi-square test.  Kruskal–Wallis for the data that did not meet the normality assumption of an ANOVA. | VAS (baseline, 3wk) | Group 1, baseline vs post-treatment p<0.000. Group 2, baseline vs post-treatment p<0.000. Group 3, baseline vs post-treatment p<0.01. | LLLT group (90 J) mean ± SD from 6.35 ± 1.18 at baseline to 4.05 ± 2.06 at 3wk.  LLLT group (45 J) mean ± SD from 5.50 ± 1.54 at baseline to 3.80 ± 1.58 at 3wk.  Placebo group (0 J) mean ± SD from 5.40 ± 1.57 at baseline to 4.55 ± 1.39 at 3wk. |
|  |  |  |  |  |  | Dosage 90 J  -0.15 (-0.78; 0.47)  Dosage 45 J  -0.39 (-1.02; 0.24) |  | SSS (baseline, 3wk) | Group 1, baseline vs post-treatment p<0.001. Group 2, baseline vs post-treatment p<0.001. Group 3, baseline vs post-treatment p<0.01. | LLLT group (90 J) mean ± SD from 23.80 ± 7.53 at baseline to 19.05 ± 8.71 at 3wk.  LLLT group (45 J) mean ± SD from 23.40 ± 6.46 at baseline to 17.70 ± 4.68 at 3wk.  Placebo group (0 J) mean ± SD from 24.65 ± 7.22 at baseline to 20.40 ± 8.40 at 3wk. |
|  |  |  |  |  |  | Dosage 90 J  -0.01 (-0.63; 0.61)  Dosage 45 J  -0.34 (-0.97; 0.28) |  | FSS (baseline, 3wk) | Group 1, baseline vs post-treatment p<0.001. Group 2, baseline vs post-treatment p<0.001. Group 3, baseline vs post-treatment p<0.05. | LLLT group (90 J) mean ± SD from 19.20 ± 6.57 at baseline to 14.60 ± 5.73 at 3wk.  LLLT group (45 J) mean ± SD from 16.25 ± 5.78 at baseline to 12.85 ± 3.54 at 3wk.  Placebo group (0 J) mean ± SD from 17.25 ± 4.89 at baseline to 14.65 ± 6.31 at 3wk. |
|  |  |  |  |  |  | Dosage 90 J  0.09 (-0.53; 0.71)  Dosage 45 J  -0.06 (-0.68; 0.56) |  | DML (baseline, 3wk) | Not significant. p-value not reported | LLLT group (90 J) mean ± SD from 4.35 ± 1.86 at baseline to 4.20 ± 1.81 at 3wk.  LLLT group (45 J) mean ± SD from 3.66 ± 0.51 at baseline to 3.98 ± 0.79 at 3wk.  Placebo group (0 J) mean ± SD from 4.19 ± 1.34 at baseline to 4.05 ± 1.35 at 3wk. |
| (Continued) | | | | | | | | | | |
| **Tascioglu et al.** | Randomized placebo-controlled, double-blind study | CTS. Not stated | Global:  46/14 | Group 1: LLLT, dosage 90 J. Gal-Al-As diode laser, power output 50 mW, wavelength 830 nm. Dose per tender joint 1.2 J, total dose per treatment 6 J, accumulated dose 90 J. 5 times per week for 3 wk; n = 20 | Group 2: LLLT, dosage 45 J. Active laser, dose per tender joint was 0.6 J, total dose per treatment 3 J, accumulated dose 45 J total. 5 times per week for 3 wk; n = 20  Group 3: placebo (0 J). 5 times per week for 3 wk; n = 20 | Dosage 90 J  0.13 (-0.49; 0.75)  Dosage 45 J  0.65 (0.01; 1.28) | One way  ANOVA to compare the differences among the three groups with the baseline.  Chi-square test.  Kruskal–Wallis for the data that did not meet the normality assumption of an ANOVA. | MNCV (baseline, 3wk) | Not significant. p-value not reported | LLLT group (90 J) mean ± SD from 57.55 ± 7.21 at baseline to 59.45 ± 5.39 at 3wk.  LLLT group (45 J) mean ± SD from 61.08 ± 4.65 at baseline to 62.32 ± 4.49 at 3wk.  Placebo group (0 J) mean ± SD from 60.34 ± 5.75 at baseline to 58.63 ± 6.53 at 3wk. |
|  |  |  |  |  |  | Dosage 90 J  -0.58 (-1.21; 0.06)  Dosage 45 J  0.00 (-0.62; 0.62) |  | SNCV - Index Finger-Wrist. (baseline, 3wk) | Not significant. p-value not reported | LLLT group (90 J) mean ± SD from 33.00 ± 5.70 at baseline to 31.53 ± 7.76 at 3wk.  LLLT group (45 J) mean ± SD from 35.89 ± 4.83 at baseline to 35.72 ± 6.73 at 3wk.  Placebo group (0 J) mean ± SD from 36.37 ± 4.26 at baseline to 35.69 ± 6.34 at 3wk. |
|  |  |  |  |  |  | Dosage 90 J  -0.58 (-1.21; 0.06)  Dosage 45 J  0.00 (-0.62; 0.62) |  | SNCV - Index Finger-Palm. (baseline, 3wk) | Group 1, baseline vs post-treatment p<0.01. Group 2, baseline vs post-treatment p<0.01. | LLLT group (90 J) mean ± SD from 25.47 ± 4.25 at baseline to 27.74 ± 3.92 at 3wk.  LLLT group (45 J) mean ± SD from 27.41 ± 4.31 at baseline to 30.94 ± 4.86 at 3wk.  Placebo group (0 J) mean ± SD from 28.09 ± 3.77 at baseline to 29.29 ± 4.60 at 3wk. |
|  |  |  |  |  |  | Dosage 90 J  -0.11 (-0.73; 0.51)  Dosage 45 J  0.29 (-0.34; 0.91) |  | Grip strength (in pounds) (baseline, 3wk) | Baseline vs. post-treatment in all groups p<0.05. | LLLT group (90 J) mean ± SD from 47.25 ± 14.37 at baseline to 52.25 ± 10.82 at 3wk.  LLLT group (45 J) mean ± SD from 53.25 ± 20.21 at baseline to 58.75 ± 17.54 at 3wk.  Placebo group (0 J) mean ± SD from 49.25 ± 13.79 at baseline to 53.85 ± 16.34 at 3wk. |

| **S5 Table.** (continued) | | | | | | | | | | |
| --- | --- | --- | --- | --- | --- | --- | --- | --- | --- | --- |
| Author | Study Design | Lesion and severity | Gender F/M | Intervention | Comparator/control | SMD | Statistical method | Outcome measures and follow-up | P-value | Outcomes |
| **Lazovic et al.** | Prospective, randomized, placebo-controlled double-blind study | CTS. Mild to moderate | G1: 36/4  G2: 34/5 | Group 1: LLLT, GaAlAs diode laser [780 nm, 30mW continuous wave, array 0.785 cm^2^, 38.2 mW/cm^2^, 90 sec/point (2.7 J, 3.4J /cm^2^/ point). Total of 20 treatments with following schedule: 10 treatments once per day, 5 days per week for 2 wk, followed by 10 treatments every other day for 3 wk;  n = 40 | Group 2: Placebo,  n = 39 | Not included in meta-analysis | Mann–Whitney U test. (between-group differences)  Wilcoxon  signed-rank test (within-group differences) | VAS (baseline, 8wk) | Between-group difference at 8wk,  p = 0.001.  Group 1, baseline vs post-treatment p<0.001. Group 2, baseline vs post-treatment p>0.05. | LLLT group: before treatment (no pain 8.20%, mild 62.30% and moderate 29.50%) vs after treatment (no pain 59%, mild 27.9% and moderate 13.1%).  Placebo group: before treatment (no pain 5.09%, mild 66.10% and moderate 28.81%) vs after treatment (no pain 10.2%, mild 71.2% and moderate 18.6%). |
|  |  |  |  |  |  | 0.52 (0.07; 0.97) | ANOVA. | SNCV (baseline, 8wk) | Group 1, baseline vs post-treatment p=0.000. Group 2, baseline vs post-treatment p=0.127. | LLLT group mean ± SD from 35.56 ± 9.48 at baseline to 41.81 ± 10.12 after treatment. Placebo group means ± SD from 35.29 ± 9.38 at baseline to 36.56 ± 9.79 after treatment. |
|  |  |  |  |  |  | -0.17 (0.61; 0.27) | ANOVA. | DML (baseline, 8wk) | Between-group difference post-treatment,  p = 0.580.  Group 1, baseline vs post-treatment p=0.000. Group 2, baseline vs post-treatment p=1.000. | LLLT group mean ± SD from 4.88 ± 1.65 at baseline to 4.69 ± 1.51 after treatment. Placebo group means ± SD from 4.99 ± 2.01 at baseline to 4.99 ± 2.01 after treatment. |

| **S5 Table.** (continued) | | | | | | | | | | |
| --- | --- | --- | --- | --- | --- | --- | --- | --- | --- | --- |
| Author | Study Design | Lesion and severity | Gender F/M | Intervention | Comparator/control | SMD | Statistical method | Outcome measures and follow-up | P-value | Outcomes |
| **Fusakul et al.** | Prospective, double-blinded randomized controlled study | CTS. Mild to moderate | G1: 54/2 (wrists)  G2: 54/2 (wrists)  66 participants 112 wrists | Group 1: LLLT. Ga-Al-As diode  Laser, wavelength 810 nm, power output of 50 mW. Dose 18 J per session. 15 sessions in total (3 times/wk for 5 weeks). Neoprene splint in neutral position for 12 weeks (day and night).  n = 56 wrists | Group 2: placebo.  Red light (wavelength of 810 nm) without laser power output. 15 sessions in total (3 times/wk for 5 weeks).  Neoprene splint in neutral for the same duration that group 1.  n = 56 wrists | 3.41 (2.82; 3.99) | Paired t-test. | VAS (baseline, 5wk, 12wk) | Between-group difference at baseline p = 0.174, at 5wk p = 0.243 and p = 0.433 at 12wk. | LLLT group mean ± SD from 6.26±0.27 at baseline to 4.25± 0.34 at 5wk to 3.45±0.38 at 12wk. Placebo group means ± SD from 4.83±0.33 at baseline to 3.15±0.30 at 5wk to 2.48±0.36 at 12wk. |
|  |  |  |  |  |  | 0.43 (0.05; 0.80) |  | SSS (baseline, 5wk, 12wk) | Between-group difference at baseline p = 0.291, at 5wk p <0.05 and p = 0.886 at 12wk. | LLLT group mean ± SD from 2.10±0.68 at baseline to 1.68±0.66 at 5wk to 1.49±0.58 at 12wk. Placebo group means ± SD from 1.68±0.56 at baseline to 1.43±0.49 at 5wk to 1.35±0.51 at 12wk. |
|  |  |  |  |  |  | 0.34 (-0.04; 0.71) |  | FSS (baseline, 5wk, 12wk) | Between-group difference at baseline p = 0.712, at 5wk p = 0.406 and p = 0.313 at 12wk. | LLLT group mean ± SD from 2.07±0.67 at baseline to 1.75±0.62 at 5wk to 1.53±0.57 at 12wk. Placebo group means ± SD from 1.77±0.62 at baseline to 1.54±0.62 at 5wk to 1.37±0.49 at 12wk. |
|  |  |  |  |  |  | -1.14 (-1.54;  -0.74) |  | DSL (baseline, 5wk, 12wk) | Between-group difference at baseline p = 0.908 and p = 0.079 at 12wk.  Group 1, baseline vs 12wk p = 0.109.  Group 2, baseline vs 12wk p = 0.166. | LLLT group mean ± SD from 4.69±0.21 at baseline to 4.48±0.13 at 12wk.  Placebo group means ± SD from 4.90±1.32 at baseline to 4.66±0.18 at 12wk. |
|  |  |  |  |  |  | 0.62 (0.24; 1.00) |  | SNAP amplitude (baseline, 5wk, 12wk) | Between-group difference at baseline p = 0.645 and  p = 0.478 at 12wk.  Group 1, baseline vs 12wk p = 0.102.  Group 2, baseline vs 12wk p = 0.350. | LLLT group mean ± SD from 24.94±1.95 at baseline to 23.00±1.74 at 12wk.  Placebo group means ± SD from 21.07±1.57 at baseline to 21.91±1.77 at 12wk. |
| (Continued) | | | | | | | | | | |

| (Continued) | | | | | | | | | | |
| --- | --- | --- | --- | --- | --- | --- | --- | --- | --- | --- |
| **Fusakul et al.** | Prospective, double-blinded randomized controlled study | CTS. Mild to moderate |  |  |  | -2.41 (-2.90; -1.92) | Paired t-test. | DML (baseline, 5wk, 12wk) | Between-group difference at baseline  p = 0.195 and  p < 0.05 at 12wk.  Group 1, baseline vs 12wk p = 0.158.  Group 2, baseline vs 12wk p = 0.194. | LLLT group mean ± SD from 4.84±0.15 at baseline to 4.73±0.13 at 12wk. Placebo group means ± SD from 5.20±0.18 at baseline to 6.63±1.10 at 12wk. |
|  |  |  |  |  |  | 0.03 (-0.34; 0.40) |  | CMAP amplitude (baseline, 5wk, 12wk) | Between-group difference at baseline  p = 0.818 and  p = 0.125 at 12wk.  Group 1, baseline vs 12wk p = 0.726.  Group 2, baseline vs 12wk p = 0.865. | LLLT group mean ± SD from 9.88±0.37 at baseline to 9.95±0.33 at 12wk.  Placebo group means ± SD from 9.90±0.36 at baseline to 9.94±0.39 at 12wk. |
|  |  |  |  |  |  | -0.55 (-0.93; -0.17) |  | Grip strength (baseline, 5wk, 12wk) | Between-group difference at baseline  p = 0.414, at 5wk  p = 0.313 and  p = 0.554 at 12wk. | LLLT group mean ± SD from 21.22±1.25 at baseline to 22.65±1.17 at 5wk to 24.49±1.15 at 12wk.  Placebo group means ± SD from 22.56±1.07 at baseline to 23.25±0.99 at 5wk to 23.60±1.00 at 12wk. |
|  |  |  |  |  |  | -1.32 (-1.73; -0.91) |  | Pinch strength (baseline, 5wk, 12wk) | Between-group difference at baseline  p = 0.169, at 5wk  p = 0.112 and  p = 0.806 at 12wk. | LLLT group mean ± SD from 4.29±0.42 at baseline to 8.00±3.56 at 5wk to 5.40±0.28 at 12wk.  Placebo group means ± SD from 4.21±0.27 at baseline to 4.65±0.30 at 5wk to 5.47±0.31 at 12wk. |

| **S5 Table.** (continued) | | | | | | | | | | |
| --- | --- | --- | --- | --- | --- | --- | --- | --- | --- | --- |
| Author | Study Design | Lesion and severity | Gender F/M | Intervention | Comparator/control | SMD | Statistical method | Outcome measures and follow-up | P-value | Outcomes |
| **Colbert et al.** | Randomized, double-blind, sham-controlled trial | CTS. Mild | G1: 15/5  G2: 16/4  G3: 15/5 | Group 1: SMF 15mT neodymium magnet that delivered 15-20mT worn during the night for 7d/week for 6wk; n=20 | Group 2: SMF 45mT neodymium magnet that delivered 45-50mT worn during the night for 7d/week for 6wk; n=20.  Group 3: sham SMF non-magnetic disk that delivered 0mT worn during the night for 7d/week for 6wk; n=20. | Dosage 15mT  0.13 (-0.49; 0.75)  Dosage 45mT 0.29 (-0.33; 0.92) | ANOVA | SSS (baseline,  6, 18wk) | Between-group comparisons of change scores from baseline to 6wk p = 0.689 and baseline to 18wk p = 0.463.  Analysis within groups: group 1 from baseline to 6wk p = 0.001 and baseline to 18wk p = 0.008; group 2 from baseline to 6wk p = 0.001 and baseline to 18wk p = 0.001; group 3 from baseline to 6wk p = 0.001 and baseline to 18wk p = 0.001. | Group 1 (15mT) mean change ± SD 0.7±0.6 from baseline to 6wk and 0.5±0.8 from baseline to 18wk.  Group 2 (45mT) mean change ± SD 0.8±0.6 from baseline to 6wk and 0.7±0.6 from baseline to 18wk.  Group 3 (0mT) mean change ± SD 0.9±0.9 from baseline to 6wk and 0.6±0.7 from baseline to 18wk. |
|  |  |  |  |  |  | Dosage 15mT  0.00 (-0.62; 0.62)  Dosage 45mT 0.19 (-0.43; 0.81) |  | FSS (baseline,  6, 18wk) | Between-group comparisons of change scores from baseline to 6wk p = 0.686, baseline to 18wk p = 0.722.  Analysis within groups: group 1 from baseline to 6wk p = 0.008 and baseline to 18wk p = 0.006; group 2 from baseline to 6wk p = 0.001 and baseline to 18wk p = 0.001; group 3 from baseline to 6wk p = 0.001 and baseline to 18wk p = 0.001 | Group 1 (15mT) mean change ± SD 0.5±0.7 from baseline to 6wk and 0.2±0.8 from baseline to 18wk.  Group 2 (45mT) mean change ± SD 0.6±0.6 from baseline to 6wk and 0.4±0.6 from baseline to 18wk.  Group 3 (0mT) mean change ± SD 0.7±0.8 from baseline to 6wk and 0.5±0.8 from baseline to 18wk. |
|  |  |  |  |  |  | Dosage 15mT  0.00 (-0.62; 0.62)  Dosage 45mT 0.52 (-0.12; 1.15) |  | DSL (baseline,  6, 18wk) | Between-group comparisons of change scores from baseline to 6wk p = 0.160, baseline to 18wk p = 0.768.  Analysis within groups: group 1 from baseline to 6wk p = 0.326 and baseline to 18wk p = 0.345; group 2 from baseline to 6wk p = 0.872 and baseline to 18wk p = 0.493; group 3 from baseline to 6wk p = 0.022 and baseline to 18wk p = 0.685. | Group 1 (15mT) mean change ± SD 0.1±0.2 from baseline to 6wk and -0.1±0.5 from baseline to 18wk.  Group 2 (45mT) mean change ± SD 0±0.4 from baseline to 6wk and -0.1±0.6 from baseline to 18wk.  Group 3 (0mT) mean change ± SD 0.2±0.3 from baseline to 6wk and 0±0.4 from baseline to 18wk. |
|  |  |  |  |  |  | Dosage 15mT  0.04 (-0.58; 0.66)  Dosage 45mT -0.26 (-0.88; 0.36) |  | SNAP amplitude (baseline,  6, 18wk) | Between-group comparisons of change scores from baseline to 6wk p = 0.734, baseline to 18wk p = 0.226.  Analysis within groups: group 1 from baseline to 6wk p = 0. 734 and baseline to 18wk p = 0. 202; group 2 from baseline to 6wk p = 0.974 and baseline to 18wk p = 0.773; group 3 from baseline to 6wk p = 0.474 and baseline to 18wk p = 0.488. | Group 1 (15mT) mean change ± SD 0.5±7 from baseline to 6wk and 2.2±7.3 from baseline to 18wk.  Group 2 (45mT) mean change ± SD 0±6 from baseline to 6wk and -0.3±4.1 from baseline to 18wk.  Group 3 (0mT) mean change ± SD 1.0±6.4 from baseline to 6wk and -1.2±7.4 from baseline to 18wk. |
| (Continued) | | | | | | | | | | |

| (Continued) | | | | | | | | | | |
| --- | --- | --- | --- | --- | --- | --- | --- | --- | --- | --- |
| **Colbert et al.** | Randomized, double-blind, sham-controlled trial | CTS. Mild | G1: 15/5  G2: 16/4  G3: 15/5 | Group 1: SMF 15mT neodymium magnet that delivered 15-20mT worn during the night for 7d/week for 6wk; n=20 | Group 3: sham SMF non-magnetic disk delivered 0mT worn during the night for 7d/week for 6wk; n=20. | Dosage 15mT  0.07 (-0.55; 0.69)  Dosage 45mT 0.00 (-0.62; 0.62) |  | DML (baseline, 6, 18wk) | Between-group comparisons of change scores from baseline to 6wk p = 0.533, baseline to 18wk p = 0.744.  Analysis within groups: group 1 from baseline to 6wk p = 0.429 and baseline to 18wk p = 0.149; group 2 from baseline to 6wk p = 0.626 and baseline to 18wk p = 0.070; group 3 from baseline to 6wk p = 0.203 and baseline to 18wk p = 0.045. | Group 1 (15mT) mean change ± SD -0.2±1.2 from baseline to 6wk and -0.3±0.8 from baseline to 18wk.  Group 2 (45mT) mean change ± SD 0.1±0.6 from baseline to 6wk and -0.1±0.3 from baseline to 18wk.  Group 3 (0mT) mean change ± SD -0.2±0.5 from baseline to 6wk and -0.3±0.5 from baseline to 18wk. |
|  |  |  |  | Group 2: SMF 45mT neodymium magnet that delivered 45-50mT worn during the night for 7d/week for 6wk; n=20. |  | Dosage 15mT  -0.34 (-0.97; 0.28)  Dosage 45mT -0.13 (-0.75; 0.49) |  | CMAP amplitude (baseline, 6, 18wk) | Between-group comparisons of change scores from baseline to 6wk p = 0.167, baseline to 18wk p = 0.049.  Analysis within groups: group 1 from baseline to 6wk p = 0.174 and baseline to 18wk p = 0.042; group 2 from baseline to 6wk p = 0.721 and baseline to 18wk p = 0.953; group 3 from baseline to 6wk p = 0.227 and baseline to 18wk p = 0.298. | Group 1 (15mT) mean change ± SD 0.2±2 from baseline to 6wk and 0±1.5 from baseline to 18wk.  Group 2 (45mT) mean change ± SD 0.8±2.5 from baseline to 6wk and 1.1±2.2 from baseline to 18wk.  Group 3 (0mT) mean change ± SD -0.6±1.9 from baseline to 6wk and -0.5±2.3 from baseline to 18wk. |

| **S5 Table.** (continued) | | | | | | | | | | |
| --- | --- | --- | --- | --- | --- | --- | --- | --- | --- | --- |
| Author | Study Design | Lesion and severity | Gender F/M | Intervention | Comparator/control | SMD | Statistical method | Outcome measures and follow-up | P-value | Outcomes |
| **Arikan et al.** | Randomized placebo-controlled, double-blind study | CTS. Mild to moderate | Global: 34/4 | Group 1: Pulsed magnetic field  therapy by using BTL-09 two-channel magneto-therapy, 30min per session,  (5 times/wk for 3wk); n=19. | Group 2: Sham therapy;  n = 19 | 0.77 (0.10; 1.43) | Wilcoxon’s test (Pre-treatment and post-treatment measures).  Mann-  Whitney U test (Between-groups if SD were higher than a half of mean) | DSL (baseline, 3wk, 7wk) | p>0.05 | Active group means difference before to after treatment means ± SD 0.22±0.31 and before treatment to 1mo follow-up 0.34±0.36. |
|  |  |  |  |  |  | 0.57 (-0.08; 1.22) |  | DML (baseline, 3wk, 7wk) | p>0.05 | Active group means difference before to after treatment means ± SD 0.32±0.52 and before treatment to 1mo follow-up 0.39±0.63.  Sham group means difference before treatment mean ± SD 0.37±0.41 and before treatment to 1mo follow-up 0.52±0.45. |
|  |  |  |  |  |  | -0.31 (-0.95; 0.33) |  | CMAP amplitude (baseline, 3wk, 7wk) | p>0.05 | Active group means difference before to after treatment means ± SD 0.03±1.87 and before treatment to 1mo follow-up -0.16±1.76.  Sham group mean difference before treatment means ± SD -0.63±2.16 and before treatment to 1mo follow-up 0.27±2.17. |
|  |  |  |  |  |  | 0.24 (-0.40; 0.87) |  | MNCV (baseline, 3wk, 7wk) | p>0.05 | Before treatment, the active group means difference was ± SD 3.48±11.43 and before treatment to 1mo follow-up 5.67±12.97.  Sham group mean difference before treatment means ± SD 1.02±5.45 and before treatment to 1mo follow-up 2.38±9.11. |

| **S5 Table.** (continued) | | | | | | | | | | | | | | | | | | | | |
| --- | --- | --- | --- | --- | --- | --- | --- | --- | --- | --- | --- | --- | --- | --- | --- | --- | --- | --- | --- | --- |
| Author | Study Design | | Lesion and severity | | Gender F/M | | Intervention | | Comparator/  control | | | SMD | Statistical method | | Outcome measures and follow-up | | P-value | | Outcomes | |
| **Wu et al.** | Prospective, randomized, controlled, double-blinded trial | | CTS. Mild to moderate | | G1: 18/2 G2: 17/3 | | Group 1: radial  extracorporeal shockwave  therapy 2000 shots, at 4-bar pressure, and a 5-Hz frequency, 1 time/wk for 3wk, plus neutral night wrist splint; n = 17. | | Group 2:  placebo radial  extracorporeal  shockwave  therapy plus  neutral night wrist splint;  n = 17 | | | -1.27 (-2.01; -0.52) | Mann–Whitney U test (differences between groups).  x^2^ test for categorical data. | | VAS (baseline, 4wk, 7wk, 11wk, 15wk) | | p<0.001 at 4wk,  p<0.001 at 7wk,  p=0.003 at 11wk,  p=0.006 at 15wk | | rESWT group means change ± SD from baseline to 4wk -3.22±0.90, baseline to 7wk -3.89±1.23, baseline to 11wk -3.59±1.49, baseline to 15wk -3.67±1.47; Placebo group mean change ± SD from baseline to 4wk -1.44±1.05, baseline to 7wk -1.79±1.19, baseline to 11wk -2.10±1.44, baseline to 15wk -2.32±1.47. | |
|  |  |  |  |  |  |  |  |  |  |  |  | -0.58 (-1.27; 0.11) |  |  | SSS (baseline, 4wk, 7wk, 11wk, 15wk) | | p=0.017 at 4wk,  p=0.005 at 7wk,  p=0.008 at 11wk,  p=0.171 at 15wk | | rESWT group means change ± SD from baseline to 4wk -12.45±8.97, baseline to 7wk -13.75±7.83, baseline to 11wk -15.15±8.58, baseline to 15wk -14.20±8.67; Placebo group mean change ± SD from baseline to 4wk -6.20±6.65, baseline to 7wk -6.50±7.42, baseline to 11wk -7.95±7.76, baseline to 15wk -10.15±9.67. | |
|  |  |  |  |  |  |  |  |  |  |  |  | -0.69 (-1.38; 0.01) |  |  | FSS (baseline, 4wk, 7wk, 11wk, 15wk) | | p=0.001 at 4wk,  p=0.002 at 7wk,  p=0.002 at 11wk,  p=0.007 at 15wk | | rESWT group means change ± SD from baseline to 4wk -5.95±.62, baseline to 7wk -6.80±3.83, baseline to 11wk -7.00±3.77, baseline to 15wk -7.10±3.60.  Placebo group means change ± SD from baseline to 4wk -2.35±2.96, baseline to 7wk -2.95±3.66, baseline to 11wk -2.70±4.28, baseline to 15wk -3.15±4.98. | |
|  |  | |  | |  | |  | |  | | | 0.10 (-0.58; 0.77) |  | | SNCV (baseline, 4wk, 7wk, 11wk, 15wk) | | p=0.362 at 4wk,  p=0.246 at 7wk,  p=0.418 at 11wk,  p=0.212 at 15wk | | rESWT group means change ± SD from baseline to 4wk 0.69±2.69, baseline to 7wk 2.24±2.45, baseline to 11wk 2.11±1.86, baseline to 15wk 3.12±2.49.  Placebo group means change ± SD from baseline to 4wk 1.36±1.78, baseline to 7wk 1.49±1.43, baseline to 11wk 1.65±1.66, baseline to 15wk 2.19±2.10. | |
|  |  | |  | |  | |  | |  | | | -0.28 (-0.96; 0.40) |  | | Pinch strength (baseline, 4wk, 7wk, 11wk, 15wk) | | p=0.852 at 4wk,  p=0.811 at 7wk,  p=0.505 at 11wk,  p=0.912 at 15wk | | rESWT group mean change ± SD from baseline to 4wk 0.90±0.68, baseline to 7wk  1.18±0.78, baseline to 11wk 1.39±0.71, baseline to 15wk 1.77±1.01.  Placebo group means change ± SD from baseline to 4wk 0.95±0.89, baseline to 7wk 1.25±0.97, baseline to 11wk 1.58±1.02, baseline to 15wk 1.74±1.08. | |
| (continued) | | | | | | | | | | | | | | | | | | | | |
| Author | | Study Design | | Lesion and severity | | Gender F/M | | Intervention | | Comparator  /control | SMD | | | Statistical method | | Outcome measures and follow-up | | P-value | | Outcomes |
| **Atya et al.** | | Randomized controlled trial | | CTS. Mild to moderate | | G1: 15/0  G2: 15/0 | | Group 1: LLLT wavelength  830Nm, power 30mw, dose per tender points 1.8 J, total dose per treatment 9 J, the accumulated dose for ten treatments 72J. Sessions of 10min/day, 2times/wk for 4 weeks,  n = 15 | | Group 2: Tendon gliding exercises. The participants were instructed about the exercises and given a brochure,  n = 15 | -1.61 (- 2.45;  -0.77) | | | A paired t-test (pre and post-treatment values of outcomes within the group)  Unpaired t-test (pre and post-treatment values of outcomes between the groups) | | VAS (baseline, after treatment) | | Analysis within groups: LLLT group from baseline to after treatment p=0.0001. TGE group from baseline to after treatment p=0.0001. | | LLLT group mean ± SD from 7.13±1.3 at baseline to 2.86±1.30 after treatment.  TGE group mean ± SD from 7.53±1.5 at baseline to 5.2±1.52 after treatment. |
|  |  |  |  |  |  |  |  |  |  |  | -1.12 (-1.9;  -0.34) | | |  |  | DSL (baseline, 4wk) | | Analysis within groups: LLLT group from baseline to after treatment p=0.0001. TGE group from baseline to after treatment p=0.0001. | | LLLT group mean ± SD from 4.44±0.49 at baseline to 3.43±0.25 after treatment.  TGE group mean ± SD from 4.01±0.22 at baseline to 3.76±0.32 after treatment. |
|  |  |  |  |  |  |  |  |  |  |  | 1.06 (0.29, 1.83) | | |  |  | SNCV (baseline, 4wk) | | Analysis within groups: LLLT group from baseline to after treatment p=0.0001. TGE group from baseline to after treatment p=0.0002. | | LLLT group mean ± SD from 34.81±1.64 at baseline to 40.81±1.67 after treatment.  TGE group mean ± SD from 35.51±0.85 at baseline to 39.07±1.52 after treatment. |
|  |  |  |  |  |  |  |  |  |  |  | -1.60 (-2.44;  -0.77) | | |  |  | DML (baseline, 4wk) | | Analysis within groups: LLLT group from baseline to after treatment p=0.0001. TGE group from baseline to after treatment p=0.0015. | | LLLT group mean ± SD from 4.53±0.35 at baseline to 3.54±0.35 after treatment.  TGE group mean ± SD from 4.86±0.3 at baseline to 4.36±0.61 after treatment. |
|  |  |  |  |  |  |  |  |  |  |  | 1.71 (0.86; 2.56) | | |  |  | Grip strength (baseline, 4wk) | | Analysis within groups: LLLT group from baseline to after treatment p=0.0001. TGE group from baseline to after treatment p=0.0001. | | LLLT group mean ± SD from 9.40±2.13 at baseline to 16.20±2.27 after treatment.  TGE group mean ± SD from 9.73±2.12 at baseline to 11.6±2.92 after treatment. |

| **S5 Table.** (continued) | | | | | | | | | | |
| --- | --- | --- | --- | --- | --- | --- | --- | --- | --- | --- |
| Author | Study Design | Lesion and severity | Gender F/M | Intervention | Comparator  /control | SMD | Statistical method | Outcome measures and follow-up | P-value | Outcomes |
| **Pratelli et al.** | Randomized controlled trial | CTS. Mild to moderate | Global: 29/13 | Group 1: LLLT wavelength  780-830Nm, power 1000-3000mW, 5 daily sessions  each 10min,  n = 35 hands | Group 2: Fascial manipulation 45min, 1 time/wk for 3wk,  n = 35 hands | 2.57 (1.93; 3.22) | Student T-test for paired data (evaluate differences in groups at T0, T1, and T2). Mann-Whitney  test for unpaired data (compare the two groups  at different times) | VAS (T0, baseline; T1, 10d after last treatment; T2, 3mo after last treatment) | Between-group  comparison at 10d and 3mo,  p<0.0001. | LLLT group mean ± SD from 5.51±2.24 at baseline to 5.00±2.07 at 10d after treatment to 5.03±2.02 at 3mo after treatment. FM group mean ± SD from 6.00±2.60 at baseline to 0.8±0.96 at 10d after treatment to 0.71±0.93 3mo after treatment. |
|  |  |  |  |  |  | 3.38 (2.64; 4.12) |  | SSS (T0, baseline; T1, 10d after last treatment; T2, 3mo after last treatment) | Between-group  comparison at 10d and 3mo,  p<0.0001. | LLLT group mean ± SD from 3.05±0.35 at baseline to 2.67±0.47 at 10d after treatment to 3.00±0.31 at 3mo after treatment. FM group mean ± SD from 3.03±0.77 at baseline to 1.36±0.27 at 10d after treatment to 1.28±0.28 3mo after treatment. |
|  |  |  |  |  |  | 1.94 (1.36; 2.51) |  | FSS (T0, baseline; T1, 10d after last treatment; T2, 3mo after last treatment) | Between-group  comparison at 10d and 3mo,  p<0.0001. | LLLT group mean ± SD from 2.90±0.89 at baseline to 2.58±0.79 at 10d after treatment to 2.63±0.94 at 3mo after treatment. FM group mean ± SD from 3.10±0.98 at baseline to 1.41±0.30 at 10d after treatment to 1.32±0.32 3mo after treatment. |

| **S5 Table.** (continued) | | | | | | | | | | |
| --- | --- | --- | --- | --- | --- | --- | --- | --- | --- | --- |
| Author | Study Design | Lesion and severity | Gender F/M | Intervention | Comparator  /control | SMD | Statistical method | Outcome measures and follow-up | P-value | Outcomes |
| **Milicin et al.** | Comparative study | Brachial plexus, Radial, Median and Ulnar palsy. | Global: 66/41 | Daily treatment scheme (same order and time): Ultrasound (0.5 W/cm^2^, 5min). Thermotherapy (fango, 40-42ºC, 15min). Electrostimulation (8min/session, increasing 1/min per session).  Kinesiotherapy (1 session/day, after that, 2 sessions/day) Massage (15 min, at the end of the therapeutic routine). All patients received 3 treatment cures, each cure of 14 days and a rest period of 3 months between the cures,  n = 107 | -- | Not included in the meta-analysis | ANOVA (the difference between  treatment phases)  Wilcoxon tests  Chi^2^  Fischer test | Muscular strength (MMT) (baseline, at the end of 3 treatment phases) | p<0.001 | Phase 1 mean ± SD from 1.16±0.70 at baseline to 2.02±0.94 at the end of treatment.  Phase 2 mean ± SD from 2.1±0.83 at baseline to 3.23±1.01 at the end of treatment.  Phase 3 mean ± SD from 3.2±0.64 at baseline to 4±0.81 at the end of treatment. |
|  |  |  |  |  |  | Not included in the meta-analysis |  | MNCV (baseline, 2,14,16,28,30wk) | p<0.001 | Phase 1 mean ± SD from 32.67±6.27 at baseline to 35.35±6.49 at the end of treatment.  Phase 2 mean ± SD from 35.47±6.88 at baseline to 39.35±7.15 at the end of treatment.  Phase 3 mean ± SD from 39.35±7.39 at baseline to 48.60±8.38 at the end of treatment. |
|  |  |  |  |  |  | Not included in the meta-analysis |  | Sensitivity (baseline, 2,14,16,28,30wk) | p<0.001 | Phase 1 mean ± SD from 9.16±2.76 at baseline to 10.24±2.98 at the end of treatment.  Phase 2 mean ± SD from 10.36±3.21 at baseline to 13.98±3.54 at the end of treatment.  Phase 3 mean ± SD from 13.98±3.37 at baseline to 16.86±3.63 at the end of treatment. |

| **S5 Table.** (continued) | | | | | | | | | | |
| --- | --- | --- | --- | --- | --- | --- | --- | --- | --- | --- |
| Author | Study Design | Lesion and severity | Gender F/M | Intervention | Comparator/control | SMD | Statistical method | Outcome measures and follow-up | P-value | Outcomes |
| **Armagan et al.** | Prospective, randomized, placebo-controlled, double-blind study | CTS. Mild to moderate | Global: 36/10 | Group 1: Splinting and continuous ultrasound therapy, frequency 1MHz,  intensity 1W/cm^2^, 1  time/day, 5 times/wk for 3wk, n=15 | Group 2: Splinting and pulsed ultrasound therapy,  frequency 1MHz,  intensity 1W/cm2, pulsed  mode duty cycle 1:4,  1 time/day, 5 times/wk for 3wk, n=16.   Group 3: Splinting and sham ultrasound  therapy 1 time/day,  5 times/wk for 3 wk, n=15 | 0.79 (0.05; 1.52) | One-way ANOVA with posthoc Tukey HSD tests and  t-tests. | VAS (baseline, 3wk) | Between-groups p=0.083 at 3wk.  Analysis within groups: group 1 from baseline to  3wk p= 0.000; group 2 p=0.041 and group 3 p= 0.003. | Continuous ultrasound group means ± SD from 5.40 ± 2.32 at baseline to 4.40±2.32 at 3wk.  Pulsed ultrasound group means ± SD from 5.56 ± 1.75 at baseline to 2.68 ± 1.92 at 3wk.  Sham ultrasound group means ± SD from 5.20 ± 1.26 at baseline to 3.53 ± 1.95 at 3wk. |
|  |  |  |  |  |  | 0.12 (-0.59; 0.82) |  | SSS (baseline, 3wk) | Between groups p=0.442 at 3wk.  Within groups: group 1 from baseline to  3wk p= 0.047; group 2 p=0.002 and group 3 p=0.001. | Continuous ultrasound group means ± SD from 26.60±8.11 at baseline to 23.06±8.13 at 3wk.  Pulsed ultrasound group means ± SD from 29.75±7.71 at baseline to 22.06±8.73 at 3wk.  Sham ultrasound group means ± SD from 25.93±4.46 at baseline to 19.66±4.60 at 3wk. |
|  |  |  |  |  |  | -0.06 (-0.76; 0.65) |  | FSS (baseline, 3wk) | Between groups p=0.125 at 3wk  Analysis within groups: group 1 from baseline to  3wk p=0.036; group 2 p=0.041 and group 3 p=0.003. | Continuous ultrasound group means ± SD from 21.33±7.37 at baseline to 18.80±7.34 at 3wk.  Pulsed ultrasound group means ± SD from 24.00±5.58 at baseline to 19.31±9.42 at 3wk.  Sham ultrasound group means ± SD from 19.00±0.85 at baseline to 14.20±4.52 at 3wk. |
|  |  |  |  |  |  | -0.29 (-1; 0.42) |  | MNCV (baseline, 3wk) | Between groups p=0.146 at 3wk | Continuous ultrasound group means ± SD from 53.82±4.10 at baseline to 54.26±4.29 at 3wk.  Pulsed ultrasound group means ± SD from 56.15±4.44 at baseline to 55.68±5.24 at 3wk.  Sham ultrasound group means ± SD from 58.05±6.43 at baseline to 57.91±5.44 at 3wk. |
| (Continued) | | | | | | | | | | |

| (Continued) | | | | | | | | | | |
| --- | --- | --- | --- | --- | --- | --- | --- | --- | --- | --- |
| **Armagan et al.** | Prospective, randomized, placebo-controlled, double-blind study | CTS. Mild to moderate | Global: 36/10 | Group 1: Splinting and continuous ultrasound therapy, frequency 1MHz,  intensity 1W/cm^2^, 1  time/day, 5 times/wk for 3wk, n=15 | Group 2: Splinting and pulsed ultrasound therapy,  frequency 1MHz,  intensity 1W/cm2, pulsed  mode duty cycle 1:4,  1 time/day, 5 times/wk for 3wk, n=16.   Group 3: Splinting and sham ultrasound  therapy 1 time/day,  5 times/wk for 3 wk, n=15 | 0.03 (-0.67; 0.74) | One-way ANOVA with posthoc Tukey HSD tests and  t-tests. | DML (baseline, 3wk) | Between groups p=0.714 at 3wk | Continuous ultrasound group mean ± SD from 4.17±0.70 at baseline to 4.19±0.82 at 3wk.  Pulsed ultrasound group means ± SD from 4.20±0.90 at baseline to 4.17±0.09 at 3wk.  Sham ultrasound group means ± SD from 4.60±1.23 at baseline to 4.45±1.37 at 3wk. |
|  |  |  |  |  |  | -0.02 (-0.73; 0.68) |  | DSL (baseline, 3wk) | Between groups p=0.912 at 3wk | Continuous ultrasound group means ± SD from 2.76±0.55 at baseline to 2.76±0.72 at 3wk.  Pulsed ultrasound group means ± SD from 2.75±0.55 at baseline to 2.65±0.62 at 3wk.  Sham ultrasound group means ± SD from 2.79±0.60 at baseline to 2.84±0.57 at 3wk. |
|  |  |  |  |  |  | 0.21 (-0.50; 0.92) |  | SNCV (baseline, 3wk) | Between groups p=0.094 at 3wk | Continuous ultrasound group means ± SD from 25.46±6.75 at baseline to 26.82±6.25 at 3wk.  Pulsed ultrasound group means ± SD from 26.28±4.17 at baseline to 29.66±6.09 at 3wk.  Sham ultrasound group means ± SD from 27.04±5.65 at baseline to 32.03±6.84 at 3wk. |

| **S5 Table.** (continued) | | | | | | | | | | |
| --- | --- | --- | --- | --- | --- | --- | --- | --- | --- | --- |
| Author | Study Design | Lesion and severity | Gender F/M | Intervention | Comparator/control | SMD | Statistical method | Outcome measures and follow-up | P-value | Outcomes |
| **Boyaci et al.** | Double-blind, randomized controlled trial | CTS. Mild to moderate | G1: 8/2 G2: 9/1  G3: 9/1 | Group 1: Continuous  shortwave diathermy  intensity position 4, 15  sessions, 20min/day, 5times/wk, 3wk and splinting, n=10 | Group 2: Pulsed shortwave  diathermy pulse duration  400ms, pulse frequency  82Hz, intensity position  6, 15 sessions, 20min/d,  5d/wk, 3wk and splinting, n=10.  Group 3: placebo plus splinting, n=10 | -0.05 (-0.93; 0.83) | Wilcoxon signed-rank test (differences between pre- and post-treatment results within the groups).  Kruskal Wallis (between groups)  Mann-  Whitney U test (post hoc comparisons) | VAS (baseline, 3wk) | p=0.315 for mean change between groups. | Continuous group mean ± SD from baseline 7.63±2.13 to 5.52±2.69 at 3wk, mean change ± SD -1.89±1.97.  Pulsed group mean ± SD from baseline 7.85±1.53 to 5.65±2.20 at 3wk, mean change ± SD -2.20±1.88.  Placebo group mean ± SD from baseline 7.56±1.50 to 6.31±0.98 at 3wk, mean change ± SD -1.25±2.46. |
|  |  |  |  |  |  | 0.07 (-0.8; 0.95) |  | SSS (baseline, 3wk) | p=0.031 for  mean change between groups, in favour of continuous group | Continuous group mean ± SD from baseline 37.10±9.59 to 29.31±8.93 at 3wk, mean change ± SD -7.78±5.23.  Pulsed group mean ± SD from baseline 32.30±9.36 to 28.65±8.38 at 3wk, mean change ± SD -3.65±4.54.  Placebo group mean ± SD from baseline 32.12±7.22 to 29.56±8.67 at 3wk, mean change ± SD -2.56±6.52. |
|  |  |  |  |  |  | 0.33 (-0.55; 1.22) |  | FSS (baseline, 3wk) | p=0.132 for  mean change between groups. | Continuous group mean ± SD from baseline 23.84±6.15 to 20.0±6.19 at 3wk, mean change ± SD -3.84±4.33.  Pulsed group mean ± SD from baseline 20.10±6.26 to 17.90±5.98 at 3wk, mean change ± SD -2.20±3.38.  Placebo group mean ± SD from baseline 18.87±6.50 to 17.68±6.61 at 3wk, mean change ± SD -1.18±2.07. |
|  |  |  |  |  |  | -0.50 (-1.39; 0.39) |  | DML (baseline, 3wk) | p=0.052 for  mean change between groups. | Continuous group mean ± SD from baseline 4.43±0.65 to 4.14±0.50 at 3wk, mean change ± SD -0.29±0.34.  Pulsed group mean ± SD from baseline 4.69±0.79 to 4.50±0.84 at 3wk, mean change ± SD -0.19±0.26.  Placebo group mean ± SD from baseline 4.37±0.53 to 4.27±0.52 at 3wk, mean change ± SD -0.10±0.17. |
| (Continued) | | | | | | | | | | |
|  |  |  |  |  |  | 0.70 (-0.21; 1.61) |  | SNCV (baseline, 3wk) | p=0.011 for  mean change between groups, in favor of continuous and pulsed group. | Continuous group mean ± SD from baseline 39.45±5.22 to 42.90±4.67 at 3wk, mean change ± SD 3.44±4.39.  Pulsed group mean ± SD from baseline 36.86±6.35 to 39.30±5.22 at 3wk, mean change ± SD 2.44±2.58.  Placebo group mean ± SD from baseline 39.01±4.89 to 39.65±5.11 at 3wk, mean change ± SD 0.63±2.24. |
|  |  |  |  |  |  | -0.64 (-1.55; 0.26) |  | DSL (baseline, 3wk) | p=0.008 for  mean change between groups, in favor of continuous and pulsed group. | Continuous group mean ± SD from baseline 2.28±0.34 to 2.15±0.29 at 3wk, mean change ± SD -0.12±0.19.  Pulsed group mean ± SD from baseline 2.60±0.64 to 2.47±0.61 at 3wk, mean change ± SD -0.13±0.14.  Placebo group mean ± SD from baseline 2.28±0.31 to 2.25±0.33 at 3wk, mean change ± SD -0.02±0.06. |

| **S5 Table.** (continued) | | | | | | | | | | |
| --- | --- | --- | --- | --- | --- | --- | --- | --- | --- | --- |
| Author | Study Design | Lesion and severity | Gender F/M | Intervention | Comparator/  control | SMD | Statistical method | Outcome measures and follow-up | P-value | Outcomes |
| **Dakowicz et al.** | Randomized controlled trial | CTS. Mild to moderate | G1: 17/1  G2: 18/2 | Group 1: LLLT. One session of Ga-As laser, (5 min and 33 sec) Physioter D-50. Pulsed  emission (200ns), wavelength 904 nm, power density 150 mW, frequency 10000 Hz, dose per point 6.0 J/cm^2^ (total dose per treatment 50 J), n=18 | Group 2: Pulsed magnetic field therapy. One session (15 min) of the PMF therapy with Magnetronic MF-10, use of sinusoidal field with a low frequency of 10 – 40 Hz, induction 1.0 - 5.0 mTesla (mT), n=20. | Not included in the meta-analysis |  | VAS (baseline, after the first 10 sessions, after 2wk break, after second 10 sessions, and six mo after the last session) | p<0.05 for both groups | Measurement scores presented in a line chart, no mean ± SD reported.  LLLT group VAS scores: 7 at the baseline, 4.4 after the first series, 2.6 after the second series, and 4.8 after 6 months.  PMF group VAS scores: 7.2 at the baseline, 4.1 after the first series, 1.8 after the second series, and 4.5 after 6 months. |

| **S5 Table.** (continued) | | | | | | | | | | |
| --- | --- | --- | --- | --- | --- | --- | --- | --- | --- | --- |
| Author | Study Design | Lesion and severity | Gender F/M | Intervention | Comparator/  control | SMD | Statistical method | Outcome measures and follow-up | P-value | Outcomes |
| **Saeed et al.** | Randomized controlled trial | CTS. Mild to moderate | G1: 31/29  G2: 35/25 (wrists) | Group 1: LLLT 9 J infrared laser diode (Enraf, Endolaser, 830 nm) at 1.8 J/point over the wrist, one session per day, 5 times a week for 4 weeks, n=50 | Group 2: Pulsed ultrasound therapy at 1MHz and intensity of 1.0 Watt/cm with Enraf Sonopuls 492, one session per day, 5 times a week for 4 weeks, n=50 | -1.78 (-2.25;  -1.32) | Student t-test. | VAS (baseline, after treatment) | p<0.001 between groups | LLLT group mean change ± SD from baseline to after treatment -2.60±1.07.  Pulsed US mean change ± SD from baseline to after treatment -4.90±1.46. |
|  |  |  |  |  |  | -1.10 (-1.52;  -0.68) |  | SSS (baseline, after treatment) | p<0.001 between groups | LLLT group mean change ± SD from baseline to after treatment -0.44±0.18.  Pulsed US mean change ± SD from baseline to after treatment -0.87±0.18. |
|  |  |  |  |  |  | -0.27 (-0.66; 0.12) |  | FSS (baseline, after treatment) | p<0.001 between groups | LLLT group mean change ± SD from baseline to after treatment -0.40±0.17.  Pulsed US mean change ± SD from baseline to after treatment -0.75±0.12. |
|  |  |  |  |  |  | 6.37 (5.39; 7.36) |  | DML (baseline, after treatment) | p<0.001 between groups | LLLT group mean change ± SD from baseline to after treatment -0.18±0.13.  Pulsed US mean change ± SD from baseline to after treatment -0.80±0.23. |
|  |  |  |  |  |  | -2.67 (-3.22;  -2.13) |  | DSL (baseline, after treatment) | p<0.001 between groups | LLLT group mean change ± SD from baseline to after treatment -0.07±0.07.  Pulsed US mean change ± SD from baseline to after treatment -0.54±0.28. |

| **S5 Table.** (continued) | | | | | | | | | | |
| --- | --- | --- | --- | --- | --- | --- | --- | --- | --- | --- |
| Author | Study Design | Lesion and severity | Gender F/M | Intervention | Comparator/  control | SMD | Statistical method | Outcome measures and follow-up | P-value | Outcomes |
| **Casale et al.** | Randomized controlled trial | CTS. Mild to moderate | G1: 5/5  G2: 5/5 | Group 1: TENS. 100 Hz (30 minutes; rectangular waves; 80 ms width, intensity below muscle contraction. One session per day, 5d/wk for 3weeks, n=10. | Group 2: LLLT. Combined 830-1064 nm Laser (radiating dose: 250 J cm^-2^ at the wrist for 100 s at 25 W (18 W [1064 nm] + 7 W [830 nm]). One session per day, 5d/wk for 3weeks, n=10 | 0.94 (0.01; 1.88) | Paired t-test (within-group comparisons).  Unpaired t-test for between-group comparisons.  Chi^2^ test for categorical variables | VAS (baseline, after treatment) | Interaction (time × group) p=0.0057.  Analysis within the groups from baseline to the end of treatment. LLLT group p=0.024; TENS group p=0.047. | TENS group mean ± SD from baseline 6.0±0.8 to 5.6±1.0 after treatment.  LLLT group mean ± SD from baseline 6.6±1.1 to 4.4±1.4 after treatment. |
|  |  |  |  |  |  | 1.24 (0.27; 2.22) |  | DML (baseline, after treatment) | Interaction (time × group) p=0.0078.  Analysis within the groups from baseline to the end of treatment. LLLT group p=0.028; TENS group p=0.15. | TENS group mean ± SD from baseline 4.8±0.6 to 4.9±0.7 after treatment.  LLLT group mean ± SD from baseline 4.5±0.6 to 4.2±0.3 after treatment. |
|  |  |  |  |  |  | -0.90 (-1.83; 0.03) |  | SNCV (baseline, after treatment) | Interaction (time × group) p=0.0019.  Analysis within the groups from baseline to the end of treatment. LLLT group p=0.014; TENS group p=0.063. | TENS group mean ± SD from baseline 33.3±4.8 to 32.4±4.7 after treatment.  LLLT group mean ± SD from baseline 33.2±5.3 to 36.3±3.5after treatment. |

| **S5 Table.** (continued) | | | | | | | | | | |
| --- | --- | --- | --- | --- | --- | --- | --- | --- | --- | --- |
| Author | Study Design | Lesion and severity | Gender F/M | Intervention | Comparator/  control | SMD | Statistical method | Outcome measures and follow-up | P-value | Outcomes |
| **Ozkan et al.** | Randomized  single-blind study | UNE. Mild to moderate | G1: 9/6  G2:9/10 | Group 1: Ultrasound treatment at a frequency  of 1 MHz and an intensity of 1.5 W/cm^2^ in continuous  mode with a transducer area of 5 cm^2^, for  5 min per session, 5 times/wk for 2 weeks, n=15 | Group 2: LLLT  wavelength  of 905 nm and mean power of 25 mW,  irradiation time of 30 s per each point (total 120 s), 5 times/wk for 2 weeks, n=17 | Not included in the meta-analysis | Mann–Whitney and Wilcoxon tests (difference pre-post treatment) | VAS (baseline, after treatment, at 1mo, at 3mo) | p=0.583 after treatment,  p=0.495 at first month,  p=0.158 in the third month. | Measurement scores presented in a line chart, no mean ± SD reported.  The US group VAS scores were 70 at the baseline, 35 after treatment, 30 in the first month, and 20 in the third month.  LLLT group VAS scores: 70 at the baseline, 30 after treatment, 30 in the first month, and 50 in the third month. |
|  |  |  |  |  |  | Not included in the meta-analysis |  | Latency change in SSCS (baseline, after treatment, at 1mo, at 3mo) | p=0.694 at first month,  p=0.221 in the third month. | US group mean ± SD from baseline 0.89±0.45 to 0.64±0.24 at first month, from baseline to 0.51± 0.21 in the third month.  LLLT group mean ± SD from baseline 0.77±0.33 to 0.61±0.17 at first month, from baseline to 0.56±0.15 at the third month. |
|  |  |  |  |  |  | -0.73 (-1.48; 0.03) |  | Handgrip force (baseline, after treatment, at 1mo, at 3mo) | p=0.084 after treatment,  p=0.088 at first month,  p=0.097 in the third month. | US group mean ± SD from baseline 0.36±0.18 to 0.48±0.2 after treatment, from baseline to 0.53±0.2 at first month, from baseline to 0.53±0.2 at the third month.  LLLT group mean ± SD from baseline 0.53±0.18 to 0.63±0.20 after treatment, from baseline to 0.67±0.21 at first month, from baseline to 0.66±0.2 in the third month. |

| **S5 Table.** (continued) | | | | | | | | | | |
| --- | --- | --- | --- | --- | --- | --- | --- | --- | --- | --- |
| Author | Study Design | Lesion and severity | Gender F/M | Intervention | Comparator/  control | SMD | Statistical method | Outcome measures and follow-up | P-value | Outcomes |
| **Paoloni et al**. | Randomized controlled trial | CTS. Mild to moderate | G1: 11/1 G2: 12/1 G3: 15/2 (wrists) | Group 1: ESWT, 4 sessions,  3wk of low intensity  focused ESWT (2500 pulses, 0.05mJ/mm^2^), n=8 (12 wrists) | Group 2: US, 15 sessions of ultrasound, 5 sessions/wk, for 3wk, 15min per session (1MHz, 1.0W/cm^2^, pulsed mode 1:4), n=8 (13 wrists).  Group 3: Cryo-US, 15 sessions of ultrasound, 5 sessions/wk, for 3wk, 15min per session (temp of 0°C, 1MHz, 1.0W/cm^2^, pulsed mode 1:4) n=9, (17 wrists) | Group 1 vs 3  0.05 (-0.91; 1.00)  Group 1 vs. 2  -0.07 (-1.05 0.91) | One-way ANOVA or Kruskal-Wallis (baseline differences).  Two-way ANOVA (between-group factor and time, within-group differences) | VAS (baseline, at the end of treatment, at 7wk, at 15wk) | Between-group  comparison for  factor treatment, p=0.72, factor  time treatment,  p=0.82. | No exact mean ± SD reported estimated values from the bar chart.    Group 1 mean ± SD from 3.85±7.40 at baseline to 2.25±5.50 after treatment to 2.00±5.00 at 7wk to 1.00±2.85 at 15wk. Group 2 from 3.90±7.40 at baseline to 1.90±4.00 after treatment to 2.10±5.00 at 7wk to 2.60±5.90 at 15wk. Group 3 from 3.45±6.60 at baseline to 1.70±4.20 after treatment to 2.80±5.90 at 7wk to 2.60±5.50 at 15wk. |
|  |  |  |  |  |  | Group 1 vs 3  0.12 (-0.86; 1.10)  Group 1 vs 2  0.00 (-0.95; 0.95) |  | SSS (baseline, at the end of treatment, at 7wk, at 15wk) | Post hoc between-group comparison  for factor treatment ESWT  group compared  with ultrasound or  cryo-ultrasound  group, p<0.05 | No exact mean ± SD reported estimated values from the bar chart.  Group 1 mean ± SD from 2.50±3.15 at baseline to 1.50±2.20 after treatment to 1.40±2.10 at 7wk to 1.25±1.50 at 15wk. Group 2 from 2.60±3.40 at baseline to 1.78±2.30 after treatment to 1.82±2.90 at 7wk and 15wk. Group 3 from 2.50±3.30 at baseline to 1.78±2.74 after treatment to 1.90±2.74 at 7wk to 1.80±2.50 at 15wk. |
|  |  |  |  |  |  | Group 1 vs 3  0.00 (-0.98; 0.98)  Group 1 vs 2  -0.05 (-1.00; 0.91) |  | FSS (baseline, at the end of treatment, at 7wk, at 15wk) | Between-group  comparison for  factor Treatment,  p>0.05, factor  time treatment,  p=0.85 | No exact mean ± SD reported estimated values from the bar chart.  Group 1 mean ± SD from 2.10±3.00 at baseline to 1.50±1.90 after treatment to 1.50±1.80 at 7wk and 15wk. Group 2 from 1.78±2.60 at baseline to 1.50±1.90 after treatment to 1.60±2.48 at 7wk and 15wk. Group 3 from 1.80±2.50 at baseline to 1.60±2.23 after treatment and 7wk to 1.50±2.10 at 15wk. |

| **S5 Table.** (continued) | | | | | | | | | | |
| --- | --- | --- | --- | --- | --- | --- | --- | --- | --- | --- |
| Author | Study Design | Lesion and severity | Gender F/M | Intervention | Comparator  /control | SMD | Statistical method | Outcome measures and follow-up | P-value | Outcomes |
| **Oshima et al**. | Non-randomized controlled trial | Radial palsy. Not stated | G1: 2/5 G2: 3/3 | Group 1: LLLT 1000 mW semiconductor laser device,  MDL 2001,  delivering 830 nm in a continuous wave, a dose of 20.1 J/cm² per point for 30 sec (5 points per session) 2times/wk for 3 months, n=7 | Group 2: LLLT + SP, same LLLT treatment combined with brace therapy, n=6 | Not included in the meta-analysis | Wilcoxon signed-rank test (pre-post treatment)  Mann-Whitney U-test (between groups) | Muscle power (MMT) (before and after treatment) | Between-groups p=0.534.  MMT score before and after treatment p=0.0015 | No exact mean ± SD was reported.  69% of patients rated LLLT treatment as effective. |

| **S5 Table.** (continued) | | | | | | | | | | |
| --- | --- | --- | --- | --- | --- | --- | --- | --- | --- | --- |
| Author | Study Design | Lesion and  severity | Gender F/M | Intervention | Comparator/  control | SMD | Statistical method | Outcome measures and follow-up | P-value | Outcomes |
| **Dincer et al.** | Prospective, randomized controlled study | CTS. Mild to moderate | G1: 36/0  G2: 30/0  G3: 34/0 (wrists) | Group 1: LLLT infrared GaAs diode laser, a wavelength of 904 nm,  the power output of 2.4 mW and a pulse frequency of 1000 Hz, 30 sec at each point (0.072 J/point at an energy density  of 1 J/cm^2^), once/day, 5times/wk for 2ws. Splinting for 3mo n=18 | Group 2: Ultrasound, at a frequency of 3 MHz and intensity  of 1.0 W/cm^2^ in continuous mode. 3min/session, once/day, 5times/wk for 2ws. Splinting for 3mo, n=15  Group 3: Splinting in a neutral position at night and during aggravating  daytime activities for 3 mo, n=17 | 0.38 (-0.33; 1.09) | MANOVA (to compare the group's profiles for all clinical and electrophysiological parameters)  ANOVA with Tukey’s HSD posthoc testing (to compare baseline values among the groups) | VAS (baseline, at 1mo and 3mo after treatment) | SpUS and SpLLL over Sp, p<0.0001 for both | Sp group mean change ± SD from 6.11±1.59 at baseline to -0.66±1.44 at 1mo to -0.67±1.75 at 3mo.  SpUS group mean change ± SD from 6.33±1.49 at baseline to -3.26±1.99 at 1mo to -3.20±2.24 at 3mo.  SpLLL group mean change ± SD from 6.22±1.35 at baseline to -3.87±1.72 at 1mo to -4.45±1.98 at 3mo. |
|  |  |  |  |  |  | 0.59 (-0.11; 1.29) |  | SSS (baseline, at 1mo and 3mo after treatment) | Between SpUS and SP group p=0.0429; between SpLLL and Sp p<0.0001; between SpUS and SpLLL p=0.03. | Sp group mean change ± SD from 3.27±0.30 at baseline to -0.18±0.17at 1mo to -0.25±0.41at 3mo.  SpUS group mean change ± SD from 3.26±0.79 at baseline to -0.52±0.51 at 1mo to -0.95±0.92 at 3mo.  SpLLL group mean change ± SD from 3.32±0.59 at baseline to -0.97±0.74 at 1mo to -1.66±0.82 at 3mo. |
|  |  |  |  |  |  | 0.65 (-0.05; 1.36) |  | FSS (baseline, at 1mo and 3mo after treatment) | SpUS and SpLLL over Sp, p<0.0001 for both.  Between SpUS and SpLLL p=0.0734. | Sp group mean change ± SD from 2.90±0.50 at baseline to -0.19±0.28 at 1mo to -0.15±0.30 at 3mo.  SpUS group mean change ± SD from 2.85±0.85 at baseline to -0.32±0.34 at 1mo to -0.80±0.37 at 3mo.  SpLLL group mean change ± SD from 2.81±0.86 at baseline to -0.64±0.66 at 1mo to -0.98±0.76 at 3mo. |
| (Continued) | | | | | | | | | | |
| **Dincer et al.** | Prospective, randomized controlled study | CTS. Mild to moderate | G1: 36/0  G2: 30/0  G3: 34/0 (wrists) | Group 1: LLLT infrared GaAs diode laser, a wavelength of 904 nm, the power output of 2.4 mW and a pulse frequency of 1000 Hz, 30 sec at each point (0.072 J/point at an energy density of 1 J/cm^2^), once/day, 5times/wk for 2ws. Splinting for 3mo n=18 | Group 2: Ultrasound, at a frequency of 3 MHz and intensity of 1.0 W/cm^2^ in continuous mode. 3min/session, once/day, 5times/wk for 2ws. Splinting for 3mo, n=15  Group 3: Splinting in a neutral position at night and during aggravating  daytime activities for 3 mo, n=17 | 0.24 (-0.44; 0.93) | MANOVA (to compare the group's profiles for all clinical and electrophysiological parameters)  ANOVA with Tukey’s HSD posthoc testing (to compare baseline values among the groups) | DML (baseline, at 1mo and 3mo after treatment) | SpUS group p=0.002, SpLLL group p<0.0001,  SpUS group p=0.0754. | Sp group mean change ± SD from 4.27±0.37 at baseline to -0.03±0.22 at 1mo to -0.03±0.29 at 3mo.  SpUS group mean change ± SD from 4.26±0.46 at baseline to -0.18±0.24 at 1mo to -0.32±0.39 at 3mo.  SpLLL group mean change ± SD from 4.25±0.45 at baseline to - 0.23±0.24 at 1mo to -0.39±0.28 at 3mo. |
|  |  |  |  |  |  | -0.37 (-1.07; 0.32) |  | SNCV (baseline, at 1mo and 3mo after treatment) | Between SpUS and SP group p=0.006; between SpLLL and Sp p<0.0001. | Sp group mean change ± SD from 38.67±4.42 at baseline to 0.79±2.13 at 1mo to 0.92±5.86 at 3mo.  SpUS group mean change ± SD from 39.81±5.10 at baseline to 3.88±4.22 at 1mo to 4.21±8.57 at 3mo.  SpLLL group mean change ± SD from 38.80±5.61at baseline to 6.57±4.53 at 1mo to 6.88±6.41 at 3mo. |

| **S5 Table.** (continued) | | | | | | | | | | |
| --- | --- | --- | --- | --- | --- | --- | --- | --- | --- | --- |
| Author | Study Design | Lesion and severity | Gender F/M | Intervention | Comparator/  control | SMD | Statistical method | Outcome measures and follow-up | P-value | Outcomes |
| **Koca et al.** | Prospective, single-blinded, randomized study | CTS. Mild to moderate | G1: 15/6  G2: 13/7  G3: 15/7 | Group 1: IFC interferential  current therapy, base frequency 4000Hz, modulation frequency  range 20Hz, delta F 10Hz and slope 1/1 in quadripolar mode, 15 sessions, 20min/session, 5 times/wk, for 3wk, n=25 | Group 3: Splinting in neutral position at night for 3wk, n=25 | 1.42 (0.72; 2.11) | Kruskal–Wallis test, Mann–Whitney U  Test, Wilcoxon test and χ^2^ test | VAS (baseline, 6wk) | Between-group 1 and group 3 p<0.01; between group 2 and group 3 p<0.01.  Between all groups  P<0.001. | Group 1 mean ± SD from 8.25±0.4 at baseline to 4.80±1.18 at 6wk; group 2 mean ± SD from 8.06±.55 at baseline to 6.68±1.42 at 6wk; group 3 mean ± SD from 8.31±0.61 at baseline to 6.37±1.18 at 6wk; |
|  |  |  |  |  |  | 0.59 (-0.04; 1.21) |  | SSS (baseline, 6wk) | Between-group 2 and group 3 p<0.05.  Between all groups  p=0.007. | Group 1 mean ± SD from 3.90±1.06 at baseline to 2.70±1.03 at 6wk; group 2 mean ± SD from 4.06±1.02 at baseline to 3.37±1.21 at 6wk; group 3 mean ± SD from 4.21±1.18 at baseline  to 3.12±1.11 at 6wk |
|  |  |  |  | Group 2: TENS pulse rate 100Hz, stimulation period 80ms, 15sessions,  20min/session, 5 times/wk, for 3wk, n=25 |  | 0.57 (-0.05; 1.2) |  | FSS (baseline, 6wk) | Between-group 2 and group 3 p<0.05.  Between all groups  p=0.031. | Group 1 mean ± SD from 2.80±1.24 at baseline to 1.90±1.21 at 6wk; group 2 mean ± SD from 2.93±1.26 at baseline to 2.50±0.78 at 6wk; group 3 mean ± SD from 3.12±1.28 at baseline  to 2.37±1.38 at 6wk. |
|  |  |  |  |  |  | 0.19 (-0.42; 0.80) |  | DML (baseline, 6wk) | Group 1 vs group 2 p<0.01; group 1 vs group 3 p<0.01. Between all groups  p=0.047. | Group 1 mean ± SD from 4.00±0.94 at baseline  to 3.89±0.88 at 6wk; group 2 mean ± SD from 4.13±0.96 at baseline to 4.06±0.88 at 6wk; group 3 mean ± SD from 4.01±0.52 at baseline to 4.06±0.61 at 6wk. |
|  |  |  |  |  |  | -0.23 (-0.85; 0.38) |  | SNCV (baseline, 6wk) | Group 1 vs group 2 p<0.05; group 1 vs group 3 p<0.05. Between all groups  p=0.010. | Group 1 mean ± SD from 40.20±2.04 at baseline to 41.80±1.76 at 6wk; group 2 mean ± SD from 39.66±1.02 at baseline to 41.38±1.78 at 6wk; group 3 mean ± SD from 39.93±1.78 at baseline to 40.75±1.48 at 6wk. |

| **S5 Table.** (continued) | | | | | | | | | | |
| --- | --- | --- | --- | --- | --- | --- | --- | --- | --- | --- |
| Author | Study Design | Lesion and severity | Gender F/M | Intervention | Comparator/  control | SMD | Statistical method | Outcome measures and follow-up | P-value | Outcomes |
| **Yagci et al.** | Prospective, randomized study | CTS. Mild to moderate | G1: 21/0 G2: 24/0 | Group 1: Full-time hand splint in neutral  position plus 10 sessions of low-level  laser therapy, n=21 | Group 2: Full-time hand splint in a neutral position for 3 mo (n=24) | -0.14 (-0.72; 0.45) | Mann–Whitney and Wilcoxon tests (between baseline and post-treatment) | SSS (baseline, 3mo) | Within-group p=0.044 for group 1 and p=0.001 for group 2. | Group 1 mean ± SD, splint: from 2.50±0.79 at baseline to 2.25±0.79 at 3mo follow-up.  Group 2 mean ± SD, splint: from 2.91±0.64 at baseline to 2.35±0.65 at 3mo follow-up. |
|  |  |  |  |  |  | -0.41 (-1.00; 0.18) |  | FSS (baseline, 3mo) | Within-group p= 0.21 for group 1 and p=0.42 for group 2. | Group 1 mean ± SD, splint: from 2.39±1.03 at baseline to 2.1±0.63 at 3mo follow-up.  Group 2 mean ± SD, splint: from 2.49±0.65 at baseline to 2.38±0.71 at 3mo follow-up. |
|  |  |  |  |  |  | 0.28 (-0.31; 0.87) |  | DML (baseline, 3mo) | Within-group p=0.003 for group 1 and p=0.3 for group 2. | Group 1 mean ± SD, splint: from 3.84±0.63 at baseline to 3.55±0.53 at 3mo follow-up.  Group 2 mean ± SD, splint: from 3.61±0.67 at baseline to 3.41±0.45 at 3mo follow-up. |
|  |  |  |  |  |  | 0.05 (-0.53; 0.64) |  | SNCV (baseline, 3mo) | Within-group p=0.001 for group 1 and p=0.194 for group 2. | Group 1 mean ± SD, splint: from 39.87±4.82 at baseline to 43.47±6.09 at 3mo follow-up.  Group 2 mean ± SD, splint: from 41.4±5.59 at baseline to 43.16±5.06 at 3mo follow-up. |

| **S5 Table.** (continued) | | | | | | | | | | |
| --- | --- | --- | --- | --- | --- | --- | --- | --- | --- | --- |
| Author | Study Design | Lesion and severity | Gender F/M | Intervention | Comparator/  control | SMD | Statistical method | Outcome measures and follow-up | P-value | Outcomes |
| **Raeissadat et al.** | Randomized clinical trial | CTS. Mild to moderate | N/R | Group 1: Bioptron group: polarized polychromatic  non-coherent light (bioptron) wavelength 480-3400Nm, power of Halogen 90W, degree of polarization 95%,  specific power density 40mW/cm^2^, energy density 2.4J/cm^2^, 8min per session, 3times/wk, 4wk, plus a neutral wrist splint for 8wk, n=27. | Group 2: Splint in a neutral position for 8wk, n=23 | 0.07 (-0.48; 0.63) | Mann-whitney-tests (between-groups comparisons). Fisher’s  exact test and Qui-two (ordinal  scales comparisons) | VAS (0, 8wk) | Between-group  difference at 8wk,  p=0.685.  Within-group p<0.05 for both. | Bioptron group mean ± SD from 6.2±1.38 at baseline to 3.78±2.19 at 8wk; control group means ± SD from 5.9±1.34 at baseline to 3.62±2.01 at 8wk. |
|  |  |  |  |  |  | Not included in the meta-analysis |  | The severity of disease (0, 8wk) | No statistical test  reported | Bioptron group severity of disease before treatment was 65.2% mild, 34.8% moderate; at 8wk 26.1% normal, 56.5% mild, and 17.4% moderate.  Control group severity of disease before treatment was 61.9% mild, 38.1% moderate; at 8wk 28.5%, 52.5% mild, 19% moderate. |
|  |  |  |  |  |  | 0.14 (-0.41; 0.7) |  | DSL (0, 8wk) | The between-group difference at 8wk,  p=0.636.  Within-group p<0.05 for both. | Bioptron group mean ± SD from 4.01±0.35 at baseline to 3.83±0.34 at 8wk; control group means ± SD from 4.01±0.37 at baseline to 3.78±0.35 at 8wk. |

| **S5 Table.** (continued) | | | | | | | | | | |
| --- | --- | --- | --- | --- | --- | --- | --- | --- | --- | --- |
| Author | Study Design | Lesion and severity | Gender F/M | Intervention | Comparator/  control | SMD | Statistical method | Outcome measures and follow-up | P-value | Outcomes |
| **Chen et al**. | Prospective, randomized, controlled, single-blinded study. | CTS. Mild to moderate | G1: 18/0  G2: 17/1 | Group1: Ultrasound-guided PRF, 1 session, 120s at 2Hz frequency, pulse width 20ms at 42°C and wrist  splint at night (8h/d) for 12wk, n=18 | Group 2: Wrist  splint at night (8h/d) for 12wk, n=18 | -1.09 (-1.79;  -0.38) | Paired t-test,  χ^2^ test, Kaplan-Meier analysis, log-rank test | VAS (1, 4, 8, 12wk after treatment) | Between-group comparisons of change scores from baseline to 1wk, p=0.002; baseline to 4wk, p=0.013; baseline to 8wk, p<0.001; baseline to 12wk, p<0.001. | PRF group mean difference ± SD from baseline to 1wk -2.9 ± 1.1 (2.4-3.4), baseline to 4wk -3.2±1.1 (2.6-3.7), baseline to 8wk -3.6 ± 1.6 (2.8-4.4), baseline to 12wk -4.2 ± 2.1 (3.2-5.2).  Splint group mean difference ± SD from baseline to 1wk -1.1 ± 1.2 (0.5-1.7), baseline to 4wk -1.6 ± 1.4 (0.9-2.2), baseline to 8wk -1.8 ± 1.5 (1.0-2.5), baseline to 12wk -2.0 ± 1.8 (1.1-2.9). |
|  |  |  |  |  |  | -0.60 (-1.27; 0.07) |  | SSS (1, 4, 8, 12wk after treatment) | Between-group comparisons of change scores from baseline to 1wk, p=.077; baseline to 4wk, P=.037; baseline  to 8wk, p=0.004; baseline to 12wk, p<0.001. | PRF group mean difference ± SD from baseline to 1wk -11.7 ± 4.7 (9.4-14.1), baseline to 4wk -13.9 ± 5.1 (11.3-16.4), baseline to 8wk -16.3 ± 6.3 (13.2-19.4), baseline to 12wk -19.7 ± 6.7 (16.4-23.0).  Splint group mean difference ± SD from baseline to 1wk -7.0 ± 6.6 (3.7-10.3), baseline to 4wk -9.4 ± 7.7 (5.6-13.3), baseline to 8wk -10.2 ± 8.7 (5.9-14.5), baseline to 12wk -10.9 ± 9.2 (6.3-15.5). |
|  |  |  |  |  |  | -0.73 (-1.41;  -0.06) |  | FSS (1, 4, 8, 12wk after treatment) | Between-group comparisons of change scores from baseline to 1wk, p=0.032; baseline to 4wk, p=0.025; baseline to 8wk, p=0.016; baseline to 12wk, p=0.001 | PRF group mean difference ± SD from baseline to 1wk -11.2 ± 4.6 (8.9-13.4), baseline to 4wk -12.1 ± 4.3 (10-14.3), baseline to 8wk -12.6 ± 5.0 (10.1-15.1), baseline to 12wk -14.2 ± 4.1 (12.2-16.3).  Splint group mean difference ± SD from baseline to 1wk -6.9 ± 6.3 (3.8-10), baseline to 4wk -8.6 ± 6.8 (5.2-11.9), baseline to 8wk -8.8 ± 7.0 (5.3-12.2), baseline to 12wk -9.3 ± 7.4 (5.6-13.0). |
|  |  |  |  |  |  | 0.40 (-0.26; 1.06) |  | Pinch strength (kg)  (1, 4, 8, 12wk after treatment) | Between-group comparisons of change scores from baseline to 1wk, p=0.282; baseline to 4wk, p=0.169; baseline to 8wk, p=0.205; baseline to 12wk, p=0.138 | PRF group mean difference ± SD from baseline to 1wk 1.0±0.6 (-1.3 to -0.7), baseline to 4wk 1.6±.8 (-2.0 to -1.2), baseline to 8wk 1.9±0.9 (-2.4 to -1.5), baseline to 12wk 2.3±1.0 (-2.8 to -1.8).  Splint group mean difference ± SD from baseline to 1wk 0.7 ± 0.6 (-1.0 to -0.4), baseline to 4wk 1.1 ± 1.2 (-1.7 to -0.5), baseline to 8wk 1.6 ± 1.0 (-2.2 to -1.1), baseline to 12wk 1.8 ± 1.2 (-2.4 to -1.2). |

| **S5 Table.** (continued) | | | | | | | | | | |
| --- | --- | --- | --- | --- | --- | --- | --- | --- | --- | --- |
| Author | Study Design | Lesion and severity | Gender F/M | Intervention | Comparator/  control | SMD | Statistical method | Outcome measures and follow-up | P-value | Outcomes |
| **Raissi et al.** | Randomized, single-blinded, controlled trial | CTS. Mild to moderate | G1: 18/2  G2: 19/1 | Group 1: rESWT, 3 sessions with 1000 shocks at a pressure of 1.5 Bar and a rate of 6pulses/s. Wrist splint in  0–5° extension, n=20 | Group 2: Wrist splint in  0–5° extension, n=20 | -0.30 (-0.92; 0.33) | Paired t-test (within-group analysis).  Independent t-test (between-groups analysis) | VAS (baseline to 3, 8, and 12 wk after treatment) | p<0.001 at 3wk, 8wk, and 12wk for both groups. | rESWT group means ± SD from 6.1±0.38 at baseline to 4.0±0.52 at 3wk; baseline to 3.25±0.56 at 8wk, baseline to 2.85±0.57 at 12wk.  Control group mean ± SD from 5.95±0.42 at baseline to 4.15±0.47 at 3wk; baseline to 3.35±0.42 at 8wk, baseline to 3.30±0.42 at 12wk. |
|  |  |  |  |  |  | -0.95 (-1.61; -0.29) |  | CMAP amplitude (baseline to 3, 8, and 12 wk after treatment) | Group 1 p=0.970 at 3wk, p=0.710 at 8wk, p=0.974 at 12wk.  Group 2 p=0.266 at 3wk, p=0.181 at 8wk, p=0.008 at 12wk. | rESWT group means ± SD from 11.76±0.88 at baseline to 11.78±0.68 at 3wk; baseline to 11.95±0.64 at 8wk, baseline to 11.78±0.61 at 12wk.  Control group mean ± SD from 12.84±0.57 at baseline to 12.41±0.62 at 3wk; baseline to 12.29±0.64 at 8wk, baseline to 11.65±0.59 at 12wk. |
|  |  |  |  |  |  | -0.59 (-1.22; 0.05) |  | SNAP amplitude (baseline to 3, 8, and 12 wk after treatment) | Group 1 p=0.909 at 3wk, p=0.994 at 8wk, p=1 at 12wk.  Group 2 p=0.125 at 3wk, p=0.695 at 8wk, p=0.865 at 12wk. | rESWT group mean ± SD from 17.68±1.99 at baseline to 17.86±2.15 at 3wk; baseline to 17.57±2.28 at 8wk, baseline to 18.73±2.57 at 12wk.  Control group mean ± SD from 20.36±2.13 at baseline to 19.11±2.01 at 3wk; baseline to 20.71±2.01 at 8wk, baseline to 20.56±1.82 at 12wk. |

| **S5 Table.** (continued) | | | | | | | | | | |
| --- | --- | --- | --- | --- | --- | --- | --- | --- | --- | --- |
| Author | Study Design | Lesion and severity | Gender F/M | Intervention | Comparator/  control | SMD | Statistical method | Outcome measures and follow-up | P-value | Outcomes |
| **Sim et al.** | Randomized, single-blinded, controlled trial | CTS. Mild to severe | G1: 23/4  G2: 26/3 | Group 1: Orthosis,  n=27 | Group 2: the combination of orthosis, nerve, and tendon gliding exercises and ultrasound  therapy, n=29 | -0.13 (-0.74;0.49) | t-tests  and chi-square tests | SSS (Before and after treatment) | P=0.8 | Orthosis group mean ± SD from 2.26 (0.8) at baseline to 1.72 (0.7) post intervention.  Combined therapy group mean ± SD 2.11(0.6) at baseline to 1.63 (0.7) post-intervention. |
|  |  |  |  |  |  | 0.06 (-0.55; 0.68) |  | FSS (Before and after treatment) | P=0.59 | Orthosis group mean ± SD from 2.05 (0.9) at baseline to 1.45 (0.4) post-intervention.  Combined therapy group mean ± SD 2.18 (0.8) at baseline to 1.48 (0.5) post-intervention. |

| **S5 Table.** (continued) | | | | | | | | | | |
| --- | --- | --- | --- | --- | --- | --- | --- | --- | --- | --- |
| Author | Study Design | Lesion and severity | Gender F/M | Intervention | Comparator/  control | SMD | Statistical method | Outcome measures and follow-up | P-value | Outcomes |
| **Ke et al.** | Randomized, controlled trial | CTS. Mild to moderate | G1: 24/6  G2: 23/6  G3: 25/5 | Group 1: one session per week of rESWT with 200 shock at a pressure of 4 Bar and 5 Hz for 3wk, n=30.  Group 2: one single session of rESWT (same conditions), n=29 | Group 3: one session of sham rESWT per week for 3, n=30 | G1: -4.48 (-5.60; -3.36)  G2: -4.85 (-6.03; -3.66) | Chi-square test/Fisher’s exact test.  One-way ANOVA.  Bonferroni post hoc | BCTQs (Baseline, 4wk, 10wk and 14wk) | *p*< 0.001 at 4wk, < 0.001 at 10wk, 0.002 at 14wk. | Group 1 of rESWT mean ± SD from 28.70 ± 1.33 at baseline. WK4-Baseline: - 11.53 ± 1.28; WK10-Baseline: -13.40 ± 1.26; WK14-Baseline: -13.30 ± 1.35.  Group 2 of rESWT mean ± SD from 24.69 ± 1.41 at baseline. WK4-Baseline: - 7.03 ± 0.93; WK10-Baseline: - 7.21 ± 1.08; WK14-Baseline: -7.86 ± 0.94. Group 3 sham rESWT mean ± SD from 26.87 ± 1.54 at baseline. WK4-Baseline: - 4.50 ± 0.98; WK10-Baseline: - 6.40 ± 1.18; WK14-Baseline: - 7.48 ± 1.35. |
|  |  |  |  |  |  | G1: -4.07 (-5.12; -3.03)  G2: -3.78 (-4.77; -2.79) |  | BCTQf (Baseline, 4wk, 10wk and 14wk) | *p*< 0.001 at 4wk, < 0.001 at 10wk, 0.002 at 14wk. | Group 1 of rESWT mean ± SD from 17.30 ± 0.96 at baseline. WK4-Baseline: - 6.80 ± 0.82; WK10-Baseline: - 7.20 ± 0.84; WK14-Baseline: -7.17 ± 0.83. Group 2 of rESWT mean ± SD from 15.17 ± 0.87 at baseline. WK4-Baseline: - 3.69 ± 0.45; WK10-Baseline: - 4.03 ± 0.46; WK14-Baseline: -4.03 ± 0.61. Group 3 sham rESWT mean ± SD from 16.30 ± 0.85 at baseline. WK4-Baseline: - 2.97 ± 0.51; WK10-Baseline: - 3.40 ± 0.70; WK14-Baseline: - 3.68 ± 0.79. |
|  |  |  |  |  |  | G1: -5.76 (-7.12; -4.40)  G2: 3.23 (2.33; 4.13) |  | SNCV (Baseline, 4wk, 10wk and 14wk) | P= 0.172 at 4wk,p= 0.760 at 10wk, p= 0.189 at 14wk. | Group 1 of rESWT mean ± SD from 31.81 ± 1.08 at baseline. WK4-Baseline: 1.04 ± 0.26; WK10-Baseline: 2.16 ± 0.42; WK14-Baseline: 3.09 ± 0.51.  Group 2 of rESWT mean ± SD from 34.17 ± 1.23 at baseline. WK4-Baseline: 1.44 ± 0.29; WK10-Baseline: 1.83 ± 0.21; WK14-Baseline: 2.50 ± 0.30.  Group 3 sham rESWT mean ± SD from 32.77 ± 1.33 at baseline. WK4-Baseline: 1.82 ± 0.33; WK10-Baseline: 1.92 ± 0.30; WK14-Baseline: 2.03 ± 0.40. |

| **S5 Table.** (continued) | | | | | | | | | | |
| --- | --- | --- | --- | --- | --- | --- | --- | --- | --- | --- |
| Author | Study Design | Lesion and severity | Gender F/M | Intervention | Comparator/  control | SMD | Statistical method | Outcome measures and follow-up | P-value | Outcomes |
| **Oztas et al.** | Randomized, controlled trial |  | G1: 7/0 G2: 9/0 G3: 9/0  * Wrists in more than one study group. | Group 1: sessions of 5 min of US with 1.5W/cm^2^ and 3MHz, 5 times per/wk for 2wk, n=7 (10 wrists).  Group 2: sessions of 5 min of US with 0.8W/cm^2^ and 3MHz, 5 times per/wk for 2wk, n=9 (10 wrists). | Group 3: sessions of 5 min of sham US (0 W/cm^2^), 5 times per/wk for 2wk, n=9 (10 wrists). | G1: -0.49 (-1.50; 0.52)  G2: -0.18 (-1.10; 0.75) | The student t-test.  One-way ANOVA | VAS pain | P>0.05 | Group 1 mean ± SD from 6.10 ± 2.50 to 2.90 ± 1.69.  Group 2 mean ± SD from 7.10 ± 2.38 to 3.60 ± 1.90.  Group 3 mean ± SD from 7.90 *±* 1.80 to 4.00 *±* 2.40. |
|  |  |  |  |  |  | G1: 0.35 (-0.65; 1.34)  G2: 0.47 (-0.47; 1.41) |  | MDL | P>0.05 | Group 1 mean ± SD from 5.85 ± 1.87 to 6.00 ± 1.95.  Group 2 mean ± SD from 5.90 ± 1.29 to 6.10 ± 1.46.  Group 3 mean ± SD from 5.60 ± 1.61 to 5.36 ± 1.48. |
|  |  |  |  |  |  | G1: 0.12 (-0.87; 1.11)  G2: -0.13 (-1.06; 0.79) |  | SDL | P>0.05 | Group 1 mean ± SD from 4.06 ± 1.39 to 3.81± 1.39.  Group 2 mean ± SD from 3.64 ± .64 to 3.53 ± .81.  Group 3 mean ± SD from 3.77 ± .89 to 3.66 ± 1.05. |
|  |  |  |  |  |  | G1: -0.18 (-1.17; 0.81)  G2: 0.51 (-0.43; 1.45) |  | SNCV | P>0.05 | Group 1 mean ± SD from 33.5 ± 8.77 to 36.6 ± 11.1.  Group 2 mean ± SD from 43.5 ± 12.1 to 45.1 ± 12.6.  Group 3 mean ± SD from 37.9 + 10.4 to 38.7 ±11.2. |

| **S5 Table.** (continued) | | | | | | | | | | |
| --- | --- | --- | --- | --- | --- | --- | --- | --- | --- | --- |
| Author | Study Design | Lesion and severity | Gender F/M | Intervention | Comparator/  control | SMD | Statistical method | Outcome measures and follow-up | P-value | Outcomes |
| **Chang et al.** | Placebo-controlled study | Mild to moderate | N/R | Group 1: LLLT (10 Hz, 50% duty cycle, 60 mW, 9.7 J/cm2, at 830 nm), 10-min session, 5 times per/wk for 2wk, n=20 | Group 2:  Sham LLLT 10-min session, 5 times per/wk for 2wk, n=20 | Not included in the meta-analysis | Mann-  Whitney U test. Wilcoxon test. Fisher’s exact test. | VAS pain (after 2wk of tmt and at 2 wk of follow-up) | P=0.001 | No exact mean ± SD reported, values presented in a bar chart. |
|  |  |  |  |  |  | -6.30 (-7.88;  -4.72) |  | SSS (after 2wk of tmt and at 2 wk of follow-up) | After 2wk of follow-up, P<0.05 | Group 1 mean ± SD from 30.80 ± 0.75 at baseline to 21.67 ± 0.58 after 2k of tmt and to 19.35 ± 0.63 at 2wk follow-up. Group 2 mean ± SD from 27.51 ± 0.50 at baseline to 25.53 ± 0.62 after 2k of tmt and 28.71 ± 0.85 at 2wk follow-up. |
|  |  |  |  |  |  | -5.78 (-7.26;  -4.31) |  | FSS (after 2wk of tmt and at 2 wk of follow-up) | After 2wk of follow-up, P<0.05 | Group 1 mean ± SD from 18.72 ± 0.84 at baseline to 13.11 ± 0.63 after 2k of tmt and to 11.04 ± 0.43 at 2wk follow-up. Group 2 mean ± SD from 18.72 ± 0.68 at baseline to 17.04 ± 0.70 after 2k of tmt and 19.60 ± 1.02 at 2wk follow-up. |
|  |  |  |  |  |  | -0.41 (-1.03; 0.22) |  | ML (after 2wk of tmt and at 2 wk of follow-up) | After 2wk of follow-up, P<0.05 | Group 1 mean ± SD from 4.23 ± 0.18 at baseline to 4.03 ± 0.33 after 2k of tmt and to 3.87 ± 0.30 at 2wk follow-up. Group 2 mean ± SD from 4.15 ± 0.20 at baseline to 4.14 ± 0.18 after 2k of tmt and 4.10 ± 0.21 at 2wk follow-up. |
|  |  |  |  |  |  | -0.35 (-0.98; 0.27) |  | SPL (after 2wk of tmt and at 2 wk of follow-up) | After 2wk of follow-up, P<0.05 | Group 1 mean ± SD from 3.92 ± 0.16 at baseline to 3.75 ± 0.21 after 2k of tmt and to 3.67 ± 0.21 at 2wk follow-up. Group 2 mean ± SD from 3.85 ± 0.13 at baseline to 3.81 ± 0.11 after 2k of tmt and 3.80 ± 0.11at 2wk follow-up. |
|  |  |  |  |  |  | 0.31 (-0.32; 0.93) |  | Grip strength (after 2wk of tmt and at 2 wk of follow-up) | After 2wk of follow-up, P<0.05 | Group 1 mean ± SD from 17.77 ± 4.37 at baseline to 19.71 ± 4.67 after 2k of tmt and to 21.19 ± 4.12 at 2wk follow-up. Group 2 mean ± SD from 18.34 ± 5.17 at baseline to 18.26 ± 4.55 after 2k of tmt and 17.38 ± 3.56 at 2wk follow-up. |

| **S5 Table.** (continued) | | | | | | | | | | |
| --- | --- | --- | --- | --- | --- | --- | --- | --- | --- | --- |
| Author | Study Design | Lesion and severity | Gender F/M | Intervention | Comparator/  control | SMD | Statistical method | Outcome measures and follow-up | P-value | Outcomes |
| **Baysal et al.** | Randomized, controlled trial | N/R | G1: 12/0  G2: 8/0  G3: 8/0  56 wrists | Group 1: received  splinting and exercise therapy (the patients were instructed to perform  nerve-and tendon-gliding exercises developed by Totten  and Hunter) 5 times per/wk for 3wk,  n = 12  Group 2:  received splinting and ultrasound therapy (15 min per session, frequency of 1 MHz and 1.0 W/cm^2^, pulsed mode 1:4), 5 times per/wk for 3wk, n = 8 | Group 3:  received splinting, exercise and ultrasound  therapy (same conditions), 5 times per/wk for 3wk, n = 8 | -0.43 (-1.24; 0.38) | Kolmogorov–  Smirnov test, Kruskal–Wallis  variance analysis and Mann–Whitney U-tests, Pearson X^2^  test | VAS pain (baseline, after the end of tmt, after 8 wk of follow-up) | p < 0.05 comparison of the before tmt and After tmt.  P < 0.05 comparison of the before tmt and After 8wk of follow-up. | Group 1 mean ± SD from 4.8 ± 2.3 at baseline to 3.3 ± 2.9 after the end of tmt and 2.6 ± 2.8 after 8wk of follow-up. Group 2 mean ± SD from 5.7 ± 2.7 at baseline to 2.2 ± 1.9 after the end of tmt and 2.5 ± 2.8 after 8wk of follow-up.  Group 3 mean ± SD from 5.6 ± 3.5 at baseline to 1.3 ± 1.8 after the end of tmt and 0.8 ± 0.9 after 8wk of follow-up. |
|  |  |  |  |  |  | -0.30 (-1.11; 0.50) |  | SSS (baseline, after the end of tmt, after 8 wk of follow-up) | p < 0.05 comparison of the before tmt and After tmt.  P < 0.05 comparison of the before tmt and After 8wk of follow-up. | Group 1 mean ± SD from 28.0 ± 9.7 at baseline to 19.7 ± 8.7 after the end of tmt and 20.2 ± 10.4 after 8wk of follow-up. Group 2 mean ± SD from 29.6 ± 9.7 at baseline to 17.1 ± 7.9 after the end of tmt and 19.1 ± 9.4 after 8wk of follow-up.  Group 3 mean ± SD from 30.4 ± 12.1 at baseline to 16.1 ± 4.8 after the end of tmt and 15.6 ± 4.7 after 8wk of follow-up. |
|  |  |  |  |  |  | 0.16 (-0.65; 0.96) |  | FSS (baseline, after the end of tmt, after 8 wk of follow-up) | p < 0.05 comparison of the before tmt and After tmt.  p < 0.05 comparison of the before tmt and After 8wk of follow-up. | Group 1 mean ± SD from 20.6 ± 7.8 at baseline to 14.8 ± 7.5 after end of tmt and 14.9 ± 6.6 after 8wk of follow-up. Group 2 mean ± SD from 21.9 ± 9.1 at baseline to 16.1 ± 8.5 after end of tmt and 16.1 ± 8.7 after 8wk of follow-up.  Group 3 mean ± SD from 20.5 ± 7.1 at baseline to 11.7 ± 3.6 after end of tmt and 12.6 ± 3.4 after 8wk of follow-up. |
| *(Continued)* | | | | | | | | | | |

| *(Continued)* | | | | | | | | | | |
| --- | --- | --- | --- | --- | --- | --- | --- | --- | --- | --- |
| **Baysal et al.** | Randomized, controlled trial | N/R | G1: 12/0  G2: 8/0  G3: 8/0  56 wrists | Group 1: received  splinting and exercise therapy (the patients were instructed to perform  nerve-and tendon-gliding exercises developed by Totten  and Hunter) 5 times per/wk for 3wk,  n = 12  Group 2:  received splinting and ultrasound therapy (15 min per session, frequency of 1 MHz and 1.0 W/cm^2^, pulsed mode 1:4), 5 times per/wk for 3wk, n = 8 | Group 3:  received splinting, exercise and ultrasound  therapy (same conditions), 5 times per/wk for 3wk, n = 8 | -0.15 (-0.95; 0.65) | Kolmogorov–  Smirnov test, Kruskal–Wallis  variance analysis and Mann–Whitney U-tests, Pearson X2 test | MDL (baseline, after end of tmt, after 8 wk of follow-up) | Not significant | Group 1 mean ± SD from 4.9 ± 1.5 at baseline to 4.8 ± 1.6 after end of tmt and to 4.8 ± 1.4 after 8wk of follow-up. Group 2 mean ± SD from 4.7 ± 1.0 at baseline to 4.6 ± 0.8 after end of tmt and 4.5 ± 0.5 after 8wk of follow-up.  Group 3 mean ± SD from 4.9 ± 1.9 at baseline to 4.6 ± 2.0 after end of tmt and 4.6 ± 2.3 after 8wk of follow-up. |
|  |  |  |  |  |  | 0.17 (-0.63; 0.97) |  | SDL (baseline, after end of tmt, after 8 wk of follow-up) | p < 0.05 comparison of the before tmt and After tmt.  p < 0.05 comparison of the before tmt and After 8wk of follow-up. | Group 1 mean ± SD from 3.5 ± 0.5 at baseline to 3.3 ± 0.4 after end of tmt and to 3.3 ± 0.5 after 8wk of follow-up. Group 2 mean ± SD from 3.4 ± 0.6 at baseline to 3.4 ± 0.7 after end of tmt and 3.3 ± 0.6 after 8wk of follow-up.  Group 3 mean ± SD from 4.0 ± 0.9 at baseline to 3.5 ± 0.6 after end of tmt and 3.5 ± 0.5 after 8wk of follow-up. |
|  |  |  |  |  |  | -0.08 (-0.88; 0.72) |  | Grip strength (baseline, after end of tmt, after 8 wk of follow-up) | p < 0.05 comparison of the before tmt and After 8wk of follow-up.  p < 0.05 comparison of the After tmt and After 8wk of follow-up | Group 1 mean ± SD from 20.5 ± 7.1 at baseline to 21.1 ± 7.0 after end of tmt and to 22.7 ± 7.4 after 8wk of follow-up. Group 2 mean ± SD from 20.6 ± 10.1 at baseline to 21.8 ± 9.7 after end of tmt and 23.5 ± 2.6 after 8wk of follow-up.  Group 3 mean ± SD from 20.7 ± 5.5 at baseline to 21.7 ± 4.9 after end of tmt and 22.3 ± 5.1 after 8wk of follow-up. |
|  |  |  |  |  |  | There were no other studies to compare in meta-analysis |  | Pinch strength (baseline, after end of tmt, after 8 wk of follow-up) | p < 0.05 comparison of the before tmt and After 8wk of follow-up.  p < 0.05 comparison of the After tmt and After 8wk of follow-up | Group 1 mean ± SD from 4.9 ± 2.5 at baseline to 5.6 ± 1.8 after end of tmt and to 6.3 ± 1.7 after 8wk of follow-up. Group 2 mean ± SD from 4.3 ± 2.2 at baseline to 5.0 ± 2.4 after end of tmt and 5.7 ± 2.3 after 8wk of follow-up.  Group 3 mean ± SD from 5.6 ± 1.4 at baseline to 6.3 ± 2.1 after end of tmt and 7.0 ± 2.2 after 8wk of follow-up. |

| **S5 Table.** (continued) | | | | | | | | | | |
| --- | --- | --- | --- | --- | --- | --- | --- | --- | --- | --- |
| Author | Study Design | Lesion and severity | Gender F/M | Intervention | Comparator/  control | SMD | Statistical method | Outcome measures and follow-up | P-value | Outcomes |
| **Bakhtiary et al.** | Randomized, controlled trial | mild to moderate | N/R  40 participants  90 wrists | Group 1: received  Ultrasound treatment (1 MHz, 1.0 W/cm2, pulse 1:4, 15 min/session) 5 times per/wk for 3wk,  n = 45 wrists | Group 2:  received low-level laser therapy (9 joules, 830 nm infrared laser at  five points),5 times per/wk for 3wk,  n = 45 wrists | 2.39 (1.65; 3.13) | Student’s t tests | VAS pain (baseline, after tmt, after 4 wk of follow-up) | p < 0.001 | Group 1 mean change ± SD -5.6 ± 1.5 from baseline to end of tmt and -6.3 ± 1.6 from baseline to 4wk.  Group 2 mean change ± SD -2.4 ± 1.2 from baseline to end of tmt and -2.0 ± 1.3 from baseline to 4wk. |
|  |  |  |  |  |  | 0.83 (0.25; 1.41) |  | MDL (baseline, after tmt, after 4 wk of follow-up) | p < 0.001 | Group 1 mean change ± SD -1.0 ± 0.6 from baseline to end of tmt and -1.1 ± 0.5 from baseline to 4wk.  Group 2 mean change ± SD -0.3 ± 0.3 from baseline to end of tmt and -0.2 ± 0.2 from baseline to 4wk. |
|  |  |  |  |  |  | There were no other studies to compare in meta-analysis |  | CMAP amplitude (baseline, after tmt, after 4 wk of follow-up) | p < 0.001 | Group 1 mean change ± SD 3.0 ± 1.6 from baseline to end of tmt and 3.6 ± 1.5 from baseline to 4wk.  Group 2 mean change ± SD 1.0 ± 2.9 from baseline to end of tmt and 1.1 ± 2.9 from baseline to 4wk. |
|  |  |  |  |  |  | -0.62 (-1.19; -0.05) |  | Index sensory latency (baseline, after tmt, after 4 wk of follow-up) | p = 0.004 | Group 1 mean change ± SD -0.8 ± 1.0 from baseline to end of tmt and -0.8 ± 1.0 from baseline to 4wk.  Group 2 mean change ± SD 0.1 ± 1.2 from baseline to end of tmt and 0.1 ± 1.1 from baseline to 4wk. |
|  |  |  |  |  |  | There were no other studies to compare in meta-analysis |  | Index SAP amplitude (baseline, after tmt, after 4 wk of follow-up) | p = 0.003 | Group 1 mean change ± SD 16.1 ± 16.4 from baseline to end of tmt and 16.8 ± 15.2 from baseline to 4wk.  Group 2 mean change ± SD 7 ± 14.2 from baseline to end of tmt and 6.5 ± 11.9 from baseline to 4wk. |
|  |  |  |  |  |  | -1.73 (-2.39;  -1.07) |  | Grip strength (baseline, after tmt, after 4 wk of follow-up) | p < 0.001 | Group 1 mean change ± SD 36.6 ± 19.1 from baseline to end of tmt and 39.3 ± 21.5 from baseline to 4wk.  Group 2 mean change ± SD 19.4 ± 15.3 from baseline to end of tmt and 21.2 ± 18.4 from baseline to 4wk. |
|  |  |  |  |  |  | There were no other studies to compare in meta-analysis |  | Pinch strength (baseline, after tmt, after 4 wk of follow-up) | p < 0.001 | Group 1 mean change ± SD 9.1 ± 4.1 from baseline to end of tmt and 9.9 ± 5.5 from baseline to 4wk.  Group 2 mean change ± SD 2.6 ± 1.0 from baseline to end of tmt and 2.9 ± 1.5 from baseline to 4wk. |

| **S5 Table.** (continued) | | | | | | | | | | |
| --- | --- | --- | --- | --- | --- | --- | --- | --- | --- | --- |
| Author | Study Design | Lesion and severity | Gender F/M | Intervention | Comparator/  control | SMD | Statistical method | Outcome measures and follow-up | P-value | Outcomes |
| **Evcik et al.** | Prospective, randomized, placebo-controlled trial | N/R | G1:33/8  G2: 37/3 | Group 1: received  laser therapy (7 joules/2 min) 5 times per/wk for 2wk,  n = 41 | Group 2: received placebo laser therapy.  5 times per/wk for 2wk,  n = 40 | Not included in the meta-analysis | One-way ANOVA.  Paired t-test. | VAS pain (before, and 4 and 12 wk after therapy) | p < 0.001 post-treatment for both groups.   Laser group p=0.006, Placebo group p > 0.05 | No exact mean ± SD reported, values presented in a bar chart. |
|  |  |  |  |  |  | Not included in the meta-analysis |  | SSS (before, and 4 and 12 wk after therapy) | p < 0.001 post-treatment for both groups.   Laser group p>0.05, Placebo group p>0.05 | No exact mean ± SD reported, values presented in a bar chart. |
|  |  |  |  |  |  | -0.11 (-0.55; 0.33) |  | MDL (before, and 4 and 12 wk after therapy) | p = 0.000 in the laser group | Group 1 mean ± SD from 4.4 ± 0.7 at baseline to 4.1 ± 0.7 after treatment.  Group 2 mean ± SD from 4.2 ± 1.00 at baseline to 4.2 ± 1.08 after treatment. |
|  |  |  |  |  |  | -0.18 (-0.62; 0.26) |  | SDL (before, and 4 and 12 wk after therapy) | p = 0.001 in the laser group | Group 1 mean ± SD from 3.2 ± 0.7 at baseline to 3.0 ± 0.5 after treatment.  Group 2 mean ± SD from 3.4 ± 1.3 at baseline to 3.1 ± 0.6 after treatment. |
|  |  |  |  |  |  | -0.08 (-0.52; 0.36) |  | Motor amplitude (baseline, after tmt, after 4 wk of follow-up) | NS | Group 1 mean ± SD from 6.8 ± 3.8 at baseline to 6.9 ± 3.4 after treatment.  Group 2 mean ± SD from 7.1 ± 3.3 at baseline to 7.2 ± 4.0 after treatment. |
|  |  |  |  |  |  | 0.13 (-0.31; 0.56) |  | Sensory amplitude (baseline, after tmt, after 4 wk of follow-up) | NS | Group 1 mean ± SD from 28.5 ± 13.4 at baseline to 29.6 ± 12.9 after treatment.  Group 2 mean ± SD from 27.6 ± 14.7 at baseline to 27.9 ± 13.4 after treatment. |
|  |  |  |  |  |  | 0.27 (-0.17; 0.71) |  | MNCV (baseline, after tmt, after 4 wk of follow-up) | NS | Group 1 mean ± SD from 51.9 ± 5.5 at baseline to 52.0 ± 6.2 after treatment.  Group 2 mean ± SD from 49.7 ± 8.1 at baseline to 50.3 ± 6.3 after treatment. |
|  |  |  |  |  |  | 0.26 (-0.18; 0.70) |  | SNCV (before, and 4 and 12 wk after therapy) | p = 0.000 in the laser group and p = 0.009 in the placebo group | Group 1 mean ± SD from 40.2 ± 8.7 at baseline to 42.9 ± 6.7 after treatment.  Group 2 mean ± SD from 39.0 ± 8.9 at baseline to 41.1 ± 7.1 after treatment. |
|  |  |  |  |  |  | 0.41 (-0.04; 0.85) |  | Grip strength (before, and 4 and 12 wk after therapy) | p = 0.005 in the laser group | Group 1 mean ± SD from 19.4 ± 6.3 at baseline to 22.4 ± 6.7 at 4wk and 22.8 ± 6.9 at 12wk. Group 2 mean ± SD from 18.0 ± 7.3 at baseline to 19.7 ± 6.5 at 4wk and 19.6 ± 7.3 at 12wk. |
|  |  |  |  |  |  | 0.57 (0.13; 1.02) |  | Pinch strength (before, and 4 and 12 wk after therapy) | p < 0.001 in the laser group and p = 0.03 in the placebo group | Group 1 mean ± SD from 4.4 ± 1.5 at baseline to 5.2 ± 1.5 at 4wk and 5.7 ± 1.6 at 12wk. Group 2 mean ± SD from 4.1 ± 1.3 at baseline to 4.6 ± 1.5 at 4wk and 4.8 ± 1.5 at 12wk. |

| **S5 Table.** (continued) | | | | | | | | | | |
| --- | --- | --- | --- | --- | --- | --- | --- | --- | --- | --- |
| Author | Study Design | Lesion and severity | Gender F/M | Intervention | Comparator/  control | SMD | Statistical method | Outcome measures and follow-up | P-value | Outcomes |
| **Carter et al.** | Double-blind placebo-controlled  randomized clinical trial | N/R | G1: 15/0  G2: 11/4 | Group 1: SMF, 1000 gauss magnet metal disk applied to the carpal tunnel area using a  Velcro wrap for a period of 45 minutes;  n = 15 | Group 2:  placebo  metal disk applied to the carpal tunnel area using a  Velcro wrap for a period of 45 minutes; n = 15 | 0.33 (-0.39; 1.06) | Chi-square, t test (paired and independent) | VAS pain (pre-tmt, at 15 min, at 30 min, post-tmt, at 2wk of follow-up) | NS for both groups | Group 1 mean ± SD from 5.9 ± 2.6 pre-treatment to 4.5 ± 2.6 at 15min to 3.7 ± 2.6 at 30min to 3.6 ± 3.1 post-treatment and 4.3 ± 2.9 at 2wk of follow-up. Group 2 mean ± SD from 5.0 ± 2.4 pre-treatment to 3.9 ± 2.8 at 15min to 3.2 ± 2.6 at 30min to 2.6 ± 2.7 post-treatment and 4.3 ± 3.5 at 2wk of follow-up. |

| **S5 Table.** (continued) | | | | | | | | | | |
| --- | --- | --- | --- | --- | --- | --- | --- | --- | --- | --- |
| Author | Study Design | Lesion and severity | Gender F/M | Intervention | Comparator/  control | SMD | Statistical method | Outcome measures and follow-up | P-value | Outcomes |
| **Weintraub and Cole.** | Randomized, double-blinded, placebo-controlled trial | Mild to severe | G1: 8/3  G2: 5/5  36 wrists | Group 1: SMF+PMF,  Participants worn a Biaxial Super Mini [Mx^2^R] device, 4 hours per/day.  n = 11 | Group 2:  Participants worn a sham Biaxial Super Mini [Mx^2^R] device, device, 4 hours per/day.  n = 10 | 0.16 (-0.70; 1.02) | ANOVA,  independent t-tests,  ANCOVA  Chi-square tests | VAS pain (baseline, after 2 months of follow-up) | NS | Group 1 mean ± SD from 6.82 ± 2.08 at baseline to 4.15 ± 2.13 at 2 months of follow-up  Group 2 mean ± SD from 5.17 ± 1.54 at baseline to 3.78 ± 2.27 at 2 months of follow-up. |
|  |  |  |  |  |  | Not included in the meta-analysis |  | CMAP amplitude (baseline, after 2 months of follow-up) |  | No exact mean ± SD reported. |
|  |  |  |  |  |  | Not included in the meta-analysis |  | SNAP (baseline, after 2 months of follow-up) |  | No exact mean ± SD reported. |

| **S5 Table.** (continued) | | | | | | | | | | |
| --- | --- | --- | --- | --- | --- | --- | --- | --- | --- | --- |
| Author | Study Design | Lesion and severity | Gender F/M | Intervention | Comparator/  control | SMD | Statistical method | Outcome measures and follow-up | P-value | Outcomes |
| **Shooshtari et al.** | Randomized, controlled trial | Mild to moderate | Total: 73/7 | Group 1: received  laser therapy (9-11 joules/cm^2^) 5 times per/wk for 3wk,  n = 40 | Group 2:  received sham laser therapy,  5 times per/wk for 3wk,  n = 40 | -8.85 (-10.33;  -7.38) | Paired t–test and independent sample t–test. | VAS pain (before, and after treatment) | p < 0.001 in both groups | Group 1 mean ± SD from 7.8 ± 042 before treatment to 4.98 ± 0.12 after treatment.  Group 2 mean ± SD from 8.01 ± 0.36 before treatment to 7.62 ± 0.4 after treatment. |
|  |  |  |  |  |  | 0.00 (-0.44; 0.44) |  | DML (before, and after treatment) | p < 0.009 in the laser group and p = 0.523 in the placebo group | Group 1 mean ± SD from 4.02 ± 0.45 before treatment to 3.94 ± 0.51 after treatment.  Group 2 mean ± SD from 3.96 ± 0.52 before treatment to 3.94 ± 0.52 after treatment. |
|  |  |  |  |  |  | -0.10 (-0.54; 0.34) |  | SL (before, and after treatment) | p < 0.001 in the laser group and p = 0.625 in the placebo group | Group 1 mean ± SD from 3.99 ± 0.46 before treatment to 3.86 ± 0.40 after treatment.  Group 2 mean ± SD from 3.91 ± 0.39 before treatment to 3.90 ± 0.39 after treatment. |
|  |  |  |  |  |  | 0.15 (-0.29; 0.59) |  | SNCV (before, and after treatment) | p < 0.001 in the laser group and p = 0.8 in the placebo group. | Group 1 mean ± SD from 32.78 ± 5.69 before treatment to 34.89 ± 5.35 after treatment.  Group 2 mean ± SD from 34.07 ± 6.30 before treatment to 34.01 ± 6.03 after treatment. |
|  |  |  |  |  |  | 0.24 (-0.20; 0.68) |  | Grip strength (before, and after treatment) | p < 0.001 in the laser group and p = 0.801 in the placebo group | Group 1 mean ± SD from 19.81 ± 5.06 before treatment to 22.86 ± 5.13 after treatment.  Group 2 mean ± SD from 21.46 ± 6.23 before treatment to 21.52 ± 6.05 after treatment. |

| **S5 Table.** (continued) | | | | | | | | | | |
| --- | --- | --- | --- | --- | --- | --- | --- | --- | --- | --- |
| Author | Study Design | Lesion and severity | Gender F/M | Intervention | Comparator/  control | SMD | Statistical method | Outcome measures and follow-up | P-value | Outcomes |
| **Badur et al.** | Randomized, controlled trial | Mild to severe | Total: 32/29 | Group 1: received  10 sessions of shortwave diathermy,5 times per/wk for 2wk plus splint  n = 31 | Group 2:  received  10 sessions of sham shortwave diathermy,5 times per/wk for 2wk plus splint  n = 30 | 0.03 (-0.48; 0.53) | Mann–Whitney U-test, Fischer’s  exact test | VAS pain (baseline, after treatment, 1 month post-treatment, 3 months post-treatment) | NS | Group 1 mean ± SD from 6.26 ± 2.72 at baseline to 5.00 ± 2.59 after treatment to 3.74 ± 2.29 at 1 month and 2.70 ± 2.23 at 3 months of follow-up.  Group 2 mean ± SD from 6.03 ± 2.75 at baseline to 4.93 ± 2.85 after treatment to 4.10 ± 2.86 at 1 month and 3.07 ± 2.60 at 3 months of follow-up. |
|  |  |  |  |  |  | 0.10 (-0.40; 0.61) |  | Grip strength (baseline, after treatment, 1 month post-treatment, 3 months post-treatment) | NS | Group 1 mean ± SD from 0.42 ± 0.19 at baseline to 0.44 ± 0.19 after treatment to 0.47 ± 0.19 at 1 month and 0.50 ± 0.21 at 3 months of follow-up.  Group 2 mean ± SD from 0.38 ± 0.22 at baseline to 0.42 ± 0.19 after treatment to 0.44 ± 0.19 at 1 month and 0.44 ± 0.19 at 3 months of follow-up. |

| **S5 Table.** (continued) | | | | | | | | | | |
| --- | --- | --- | --- | --- | --- | --- | --- | --- | --- | --- |
| Author | Study Design | Lesion and severity | Gender F/M | Intervention | Comparator/  control | SMD | Statistical method | Outcome measures and follow-up | P-value | Outcomes |
| **Jothi and Bland** | Randomized, double blind, single-center | Mild to moderate | G1: 17/3  G2: 14/6 | Group 1: received pulsed mode ultrasound  treatment (1 MHz, 1.0 W/cm^2^, 15 min/session) 5 times per/wk for the first 2wk, then 2 times per/wk for 5wk and a night splint  n = 20 | Group 2: received sham ultrasound  treatment 5 times per/wk for the first 2wk, then 2 times per/wk for 5wk and a night splint  n = 20 | -0.15 (-0.77; 0.47) | Mann–Whitney U tests and Fisher's exact test | SSS (baseline, after 7 wk, 6 and 12 months of follow-up) | p<0.05 in both groups at 7wk, 6 and 12 months.  p<0.001 in the placebo group at 6 months. | Group 1 mean change ± SD 2.00 ± 0.72 from baseline to 7wk, 1.79 ± 0.83 from baseline to 6mo and 1.82 ± 0.70 from baseline to 12mo.  Group 2 mean change ± SD 2.11 ± 0.74 from baseline to 7wk, 1.77 ± 0.71 from baseline to 6mo and 1.68 ± 0.85 from baseline to 12mo. |
|  |  |  |  |  |  | 0.13 (-0.49; 0.75) |  | FSS (baseline, after 7 wk, 6 and 12 months of follow-up) | NS | Group 1 mean change ± SD 1.65 ± 0.73 from baseline to 7wk, 1.56 ± 0.82 from baseline to 6mo and 1.57 ± 0.73 from baseline to 12mo.  Group 2 mean change ± SD 1.85 ± 0.71 from baseline to 7wk, 1.69 ± 0.79 from baseline to 6mo and 1.46 ± 0.70 from baseline to 12mo. |
|  |  |  |  |  |  | 0.13 (-0.49; 0.75) |  | DML (baseline, after 7 wk, 6 and 12 months of follow-up) | NS | Group 1 mean change ± SD 4.3 ± 0.8 from baseline to 7wk, 4.1 ± 0.9 from baseline to 6mo and 4.0 ± 0.6 from baseline to 12mo.  Group 2 mean change ± SD 4.2 ± 0.7 from baseline to 7wk, 4.0 ± 0.5 from baseline to 6mo and 3.8 ± 0.4 from baseline to 12mo |
|  |  |  |  |  |  | -0.21 (-0.83; 0.41) |  | SNCV (baseline, after 7 wk, 6 and 12 months of follow-up) | p<0.05 in placebo group at 7wk;  p<0.05 in both groups at 6 and 12 months | Group 1 mean change ± SD 40.2 ± 10.9 from baseline to 7wk, 43.6 ± 5.8 from baseline to 6mo and 44.1 ± 5.4 from baseline to 12mo.  Group 2 mean change ± SD 42.2 ± 7.4 from baseline to 7wk, 43.7 ± 5.3 from baseline to 6mo and 45.6 ± 3.9 from baseline to 12mo |

| **S5 Table.** (continued) | | | | | | | | | | |
| --- | --- | --- | --- | --- | --- | --- | --- | --- | --- | --- |
| Author | Study Design | Lesion and severity | Gender F/M | Intervention | Comparator/  control | SMD | Statistical method | Outcome measures and follow-up | P-value | Outcomes |
| **Wolny et al.** | Randomized, controlled trial | Mild to moderate | G1:62/8  G2: 60/10 | Group 1: Electrophysical modalities group; received Laser (started with a red laser 658-nm light at 50 mW, for 1’40’’, dose of 5 J. Next, an infrared laser 808-nm light at 400  mW, for 1min and a dose of 24 J) and  Ultrasound treatment (1 MHz, 1.0 W/cm, pulse width factor of 75%).  Each procedure lasted 15min, 2 times per/wk for 5wk,  n = 70 | Group 2: Manual therapy group;  received neurodynamic techniques, functional massage, and carpal bone mobilizations techniques, 2 times per/wk for 5wk,  n = 70 | 1.23 (0.87; 1.59) | Independent t test, χ^2^ test  One way ANOVA, Bonferroni’s post hoc test | SSS (pre-treatment and pos-treatment) | P <.01 in both groups | Group 1 mean ± SD from 2.94 ± 0.74 before treatment to 2.57 ± 0.77 after treatment.  Group 2 mean ± SD from 2.97 ± 0.63 before treatment to 1.78 ± 0.47 after treatment. |
|  |  |  |  |  |  | 0.81 (0.46; 1.15) |  | FSS (pre-treatment and pos-treatment) | P <.01 in both groups | Group 1 mean ± SD from 2.77 ± 0.94 before treatment to 2.55 ± 0.95 after treatment.  Group 2 mean ± SD from 2.80 ± 0.94 before treatment to 1.90 ± 0.62 after treatment. |
|  |  |  |  |  |  | 0.19 (-0.14; 0.52) |  | ML (pre-treatment and pos-treatment) | P <.01 in the MT group;  P = .001 in the EM group | Group 1 mean ± SD from 5.45 ± 1.12 before treatment to 5.24 ± 1.17 after treatment.  Group 2 mean ± SD from 5.61 ± 1.08 before treatment to 5.02 ± 1.13 after treatment. |
|  |  |  |  |  |  | There were no other studies to compare in meta-analysis |  | SL (pre-treatment and pos-treatment) | P <.01 in the MT group;  P = .05 in the EM group | Group 1 mean ± SD from 1.10 ± 0.18 before treatment to 1.06 ± 1.18 after treatment.  Group 2 mean ± SD from 1.15 ± 0.16 before treatment to 1.01 ± 0.17 after treatment. |
|  |  |  |  |  |  | 0.34 (0.01; 0.68) |  | SNCV (pre-treatment and pos-treatment) | P <.01 in the MT group | Group 1 mean ± SD from 38.2 ± 11.1 before treatment to 39.22 ± 11.91 after treatment.  Group 2 mean ± SD from 26.2 ± 15.7 before treatment to 35.1 ± 12.1 after treatment. |
|  |  |  |  |  |  | There were no other studies to compare in meta-analysis |  | MNCV (pre-treatment and pos-treatment | P <.01 in the MT group | Group 1 mean ± SD from 54.8 ± 5.6 before treatment to 55.3 ± 5.7 after treatment.  Group 2 mean ± SD from 53.2 ± 7.8 before treatment to 56.5 ± 7.8 after treatment. |
