# Supplementary material for: Effectiveness of electrophysical modalities in the sensorimotor rehabilitation of radial, ulnar, and median neuropathies: A meta-analysis
Source: PLoS One. 2021 Mar 18;16(3):e0248484. doi: 10.1371/journal.pone.0248484 (PMC7971482; doi:10.1371/journal.pone.0248484)
Supplement: S1 File — (PDF) [file pone.0248484.s006.pdf]

Effectiveness of electrotherapy modalities in the sensorimotor rehabilitation of radial, ulnar and median neuropathies: a systematic review

Ena Bula Oyola, Juan Manuel Belda Lois, Rosa María Porcar Seder, Álvaro Page Del Pozo

To enable PROSPERO to focus on COVID-19 registrations during the 2020 pandemic, this registration record was automatically published exactly as submitted. It has not been checked for eligibility or for sense by the PROSPERO team.

Citation

Ena Bula Oyola, Juan Manuel Belda Lois, Rosa María Porcar Seder, Álvaro Page Del Pozo. Effectiveness of electrotherapy modalities in the sensorimotor rehabilitation of radial, ulnar and median neuropathies: a systematic review. PROSPERO 2020 CRD42020168792 Available from: https://www.crd.york.ac.uk/prospero/display_record.php?ID=CRD42020168792

Review question

In patients with radial, ulnar y/o median neuropathy are electrophysical therapies more effective than standard treatments in sensorimotor rehabilitation?

Searches

The search was carried out between April and July 2019 using medical topic headings (MeSH) and free text terms for neuropathies and rehabilitation in the Cochrane and PubMed databases. Only studies in English that were conducted in the last 10 years were included.

Types of study to be included

We will include randomised control trials (RCTs), controlled clinical trials (non-randomised), systematic reviews and meta-analyses. We will include articles reported in the English language from the last 10 years.

Condition or domain being studied

Peripheral nerves can be damaged by autoimmune or metabolic disorders, tumors, or by thermal, chemical, or mechanical trauma. The most common injuries are penetrating, crush or pull injuries and ischemia. Most involve the upper limbs, with a higher rate of involvement of the ulnar nerve, followed by the median and radial. Symptoms may include neuromuscular affectations: partial or total motor dysfunction of the forearm and hand, loss of muscle tone and strength; and neurosensorial alterations: hypesthesia or hyperesthesia, pain, allodynia and/or paresthesia. The recovery responds to factors such as age, type of nerve affected, origin and type of injury. The therapeutic treatment seeks the relief of symptoms and neuromuscular conservation through physical therapy, electrophysical therapy and the use of orthotics.

Participants/population

Adults with radial, median and/or ulnar neuropathy whose treatment includes electrophysical therapy. Children and adolescents or patients with neuropathies of degenerative, endocrine or metabolic origin will be excluded.

Intervention(s), exposure(s)

Provision of treatments with electrophysical therapies (Low-level laser therapy, ultrasound, magnetotherapy, radial extracorporeal shockwave, etc.)

Comparator(s)/control

Non-provision of treatments with electrophysical therapies, placebo or provision of treatment with manual physical therapy.

Context

Main outcome(s)

Improvement of neuropathic symptoms:

Symptoms severity (BQSSS: Boston Questionnaire symptom severity scale, DASH, Quick DASH)

Functional status (BQFSS: Boston Questionnaire functional status scale, OPUS: Upper extremity functional status, Abilhand, MHO: Michigan Hand Outcomes Questionnaire)

Pain score (visual analog scale (VAS), Neuropathic pain scale (NPS) or any validated instrument)

Sensitive state (Surface tactile testing - 2 point discrimination / RASP)

* Measures of effect

Minimum 1 month follow-up

Additional outcome(s)

Sex, age, clinical symptoms, and nerve conduction study results. Safety of electrophysical modalities and application parameters.

* Measures of effect

Minimum 1 month follow-up

Data extraction (selection and coding)

Two reviewers will independently examine the titles and abstracts resulting from the search against the eligibility criteria, to reduce the likelihood of error. We will obtain full reports of all titles that appear to meet the inclusion criteria. Any dispute over eligibility between reviewers will be resolved through discussion with a third reviewer. Subsequently, both reviewers will independently review the full-text articles to assess whether they meet the eligibility criteria. Any disagreement on study eligibility between reviewers will be resolved through discussion with a third reviewer.

We will carry out a global mapping of the studies through a flow chart according to the PRISMA methodology. We will develop a data extraction matrix that gathers the sources consulted, type of study, keywords and the inclusion and exclusion criteria. The extracted studies will be classified in a matrix that will include type of nerve studied, type of lesion, severity, characteristics of the participants (number, age, sex), follow-up period, description of experimental and comparative interventions, type of study, main results and conclusions. Data will be extracted by one reviewer and then examinated by two other reviewers.

Risk of bias (quality) assessment

The quality assessment of the included studies will be carried out by two reviewers independently, and any disagreement will be resolved by discussion in the presence of a third reviewer. We will assess the risk of bias in the included studies using the risk of bias tool included in the Cochrane Handbook for Systematic Reviews of Interventions.

Strategy for data synthesis

The results of the trials will be combined in a meta-analysis where possible. Otherwise, we will produce a descriptive synthesis of the individual results.

Analysis of subgroups or subsets

If possible, we will make subgroups with the data according to the pathology, age and type of treatment.

Contact details for further information

Ena Bula Oyola
oyolae@uninorte.edu.co

Organisational affiliation of the review

Universitat Politècnica de València and Universidad del Norte
http://www.upv.es/ and https://www.uninorte.edu.co/

Review team members and their organisational affiliations

Ena Bula Oyola. Universidad del Norte, Universitat Politècnica de València
Dr Juan Manuel Belda Lois. Instituto de Biomecánica de Valencia, Universitat Politècnica de València
Dr Rosa María Porcar Seder. Instituto de Biomecánica de Valencia
Dr Álvaro Page Del Pozo. Instituto de Biomecánica de Valencia, Universitat Politècnica de València

Type and method of review

Systematic review

Anticipated or actual start date

01 April 2019

Anticipated completion date

14 March 2020

Funding sources/sponsors

Ena Bula is the beneficiary of the Colciencias scholarship "Formación de capital humano de alto nivel para las regiones - Atlántico" and the teacher training program of the Universidad del Norte in Colombia.

Conflicts of interest

Language

English

Country

Colombia, Spain

Stage of review

Review Ongoing

Subject index terms status

Subject indexing assigned by CRD

Subject index terms

MeSH headings have not been applied to this record

Date of registration in PROSPERO

28 April 2020

Date of publication of this version

28 April 2020

Details of any existing review of the same topic by the same authors

Stage of review at time of this submission

Stage	Started	Completed
Preliminary searches	Yes	Yes
Piloting of the study selection process	Yes	Yes
Formal screening of search results against eligibility criteria	Yes	Yes
Data extraction	No	No
Risk of bias (quality) assessment	No	No
Data analysis	No	No

The record owner confirms that the information they have supplied for this submission is accurate and complete and they understand that deliberate provision of inaccurate information or omission of data may be construed as scientific misconduct.

The record owner confirms that they will update the status of the review when it is completed and will add publication details in due course.

Versions

28 April 2020

PROSPERO
This information has been provided by the named contact for this review. CRD has accepted this information in good faith and registered the review in PROSPERO. The registrant confirms that the information supplied for this submission is accurate and complete. CRD bears no responsibility or liability for the content of this registration record, any associated files or external websites.
