# Supplementary material for: Effectiveness of electrophysical modalities in the sensorimotor rehabilitation of radial, ulnar, and median neuropathies: A meta-analysis
Source: PLoS One. 2021 Mar 18;16(3):e0248484. doi: 10.1371/journal.pone.0248484 (PMC7971482; doi:10.1371/journal.pone.0248484)
Supplement: S2 File — (DOCX) [file pone.0248484.s007.docx]

# **S2 File. Search terms**

# Search terms

## Cochrane and Pubmed

## Keywords:

| *Radial nerve neuropathy*  *OR radial palsy*  *OR sensorimotor alterations*  *NOT pharmacological treatment*  *NOT surgical treatment* | *Ulnar nerve neuropathy*  *OR ulnar palsy*  *OR sensorimotor alterations*  *NOT pharmacological treatment*  *NOT surgical treatment* | *Median nerve neuropathy*  *OR median palsy*  *OR sensorimotor alterations*  *NOT pharmacological treatment*  *NOT surgical treatment* |
| --- | --- | --- |
| *Radial nerve injury*  *OR radial palsy*  *OR sensory dysfunction*  *Or motor dysfunction*  *NOT pharmacological treatment* | *Ulnar nerve injury*  *OR ulnar palsy*  *OR sensory dysfunction*  *Or motor dysfunction*  *NOT pharmacological treatment* | *Median nerve injury*  *OR median palsy*  *OR sensory dysfunction*  *Or motor dysfunction*  *NOT pharmacological treatment* |
| *Radial nerve neuropathy*  *OR radial palsy*  *OR sensitive alteration*  *NOT pharmacological treatment*  *NOT surgical treatment* | *Ulnar nerve neuropathy*  *OR ulnar palsy*  *OR sensorimotor alterations*  *NOT pharmacological treatment*  *NOT surgical treatment* | *Median nerve neuropathy*  *OR median palsy*  *OR sensorimotor alterations*  *NOT pharmacological treatment*  *NOT surgical treatment* |

## PubMed MESH:

(("Radial Neuropathy/rehabilitation"[Mesh] OR "Radial Neuropathy/therapy"[Mesh])) OR ("Median Neuropathy/rehabilitation"[Mesh] OR "Median Neuropathy/therapy"[Mesh]) OR ("Ulnar Neuropathies/rehabilitation"[Mesh] OR "Ulnar Neuropathies/therapy"[Mesh]))

((((median neuropathy) OR (ulnar neuropathy)) OR (radial neuropathy)) AND (electrophysical)) AND (treatment)

SEMANTIC SCHOLAR

radial neuropathy median neuropathy ulnar neuropathy electrophysical modality

PEDro


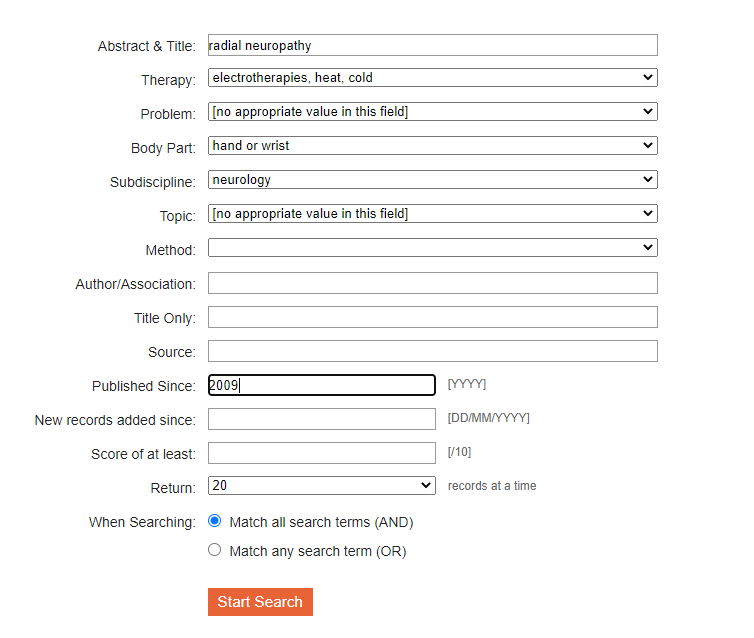


Radial nerve palsy

LILACS

|  |
| --- |

| \| Base de datos : \| **LILACS** \| \| --- \| --- \| \| Búsqueda : \| **paralisis radial [Palabras] and electrofisica [Palabras] and not farmaco [Palabras]** \| |
| --- | --- | --- | --- | --- |

| Base de datos : | **LILACS** |
| --- | --- |
| Búsqueda : | **neuropatia radial [Palabras] and electrofisica [Palabras] and not farmaco [Palabras]** |
| Referencias encontradas : | **0** |

|  |
| --- |

| \| Base de datos : \| **LILACS** \| \| --- \| --- \| \| Búsqueda : \| **paralisis cubital [Palabras] and electrofisica [Palabras] and not farmaco [Palabras]** \| \| Referencias encontradas : \| **0** \| |
| --- | --- | --- | --- | --- | --- | --- |

|  |
| --- |

| \| Base de datos : \| **LILACS** \| \| --- \| --- \| \| Búsqueda : \| **neuropatia cubital [Palabras] and electrofisica [Palabras] and not farmaco [Palabras]** \| \| Referencias encontradas : \| **0** \| |
| --- | --- | --- | --- | --- | --- | --- |

| \| Base de datos : \| **LILACS** \| \| --- \| --- \| \| Búsqueda : \| **neuropatia mediana [Palabras] and electrofisica [Palabras] and not farmaco [Palabras]** \| \| Referencias encontradas : \| **0** \| |
| --- | --- | --- | --- | --- | --- | --- |

|  |
| --- |

| \| Base de datos : \| **LILACS** \| \| --- \| --- \| \| Búsqueda : \| **paralisis nervio mediano [Palabras] and electrofisica [Palabras] and not farmaco [Palabras]** \| \| Referencias encontradas : \| **0** \| |
| --- | --- | --- | --- | --- | --- | --- |

**WEB OF SCIENCE**

You searched for: (TS=electrophysical modalities) AND LANGUAGE: (English)

Timespan: All years. Indexes: SCI-EXPANDED, SSCI, A&HCI, CPCI-S, BKCI-S, BKCI-SSH, ESCI.

You searched for: (TS=(radial neuropathy OR median neuropathy OR ulnar neuropathy) AND TS=treatment) AND LANGUAGE: (English)

Timespan: All years. Indexes: SCI-EXPANDED, SSCI, A&HCI, CPCI-S, BKCI-S, BKCI-SSH, ESCI.

**SCOPUS**

Radial neuropathy OR median neuropathy OR ulnar neuropathy AND electrophysical AND NOT surgical AND NOT pharmacological

Radial nerve OR median nerve OR ulnar nerve AND electrophysical therapy AND NOT surgical AND NOT pharmacological

**EBSCOhost**


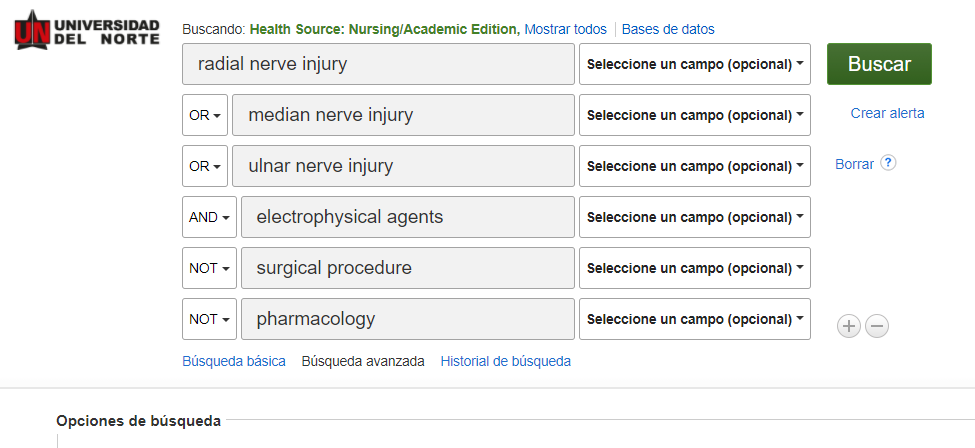


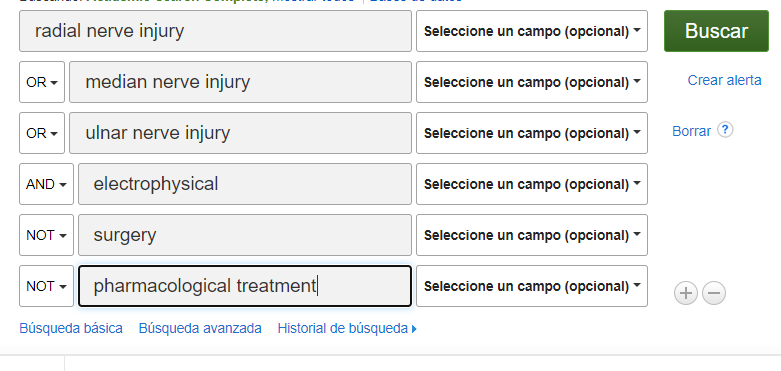


**OVID**

Radial neuropathy OR median neuropathy OR ulnar neuropathy AND electrophysical AND NOT surgical AND NOT pharmacological

**BIOMED CENTRAL (BMC)**

Electrophysical agents

Radial nerve injury + electrophysical

Median nerve injury + electrophysical

Ulnar nerve injury + electrophysical

Radial neuropathy + electrophysical

Median neuropathy + electrophysical

Ulnar neuropathy electrophysical
