# Supplementary material for: Effectiveness of electrophysical modalities in the sensorimotor rehabilitation of radial, ulnar, and median neuropathies: A meta-analysis
Source: PLoS One. 2021 Mar 18;16(3):e0248484. doi: 10.1371/journal.pone.0248484 (PMC7971482; doi:10.1371/journal.pone.0248484)
Supplement: Attachment — Submitted filename: Response to Reviewers.doc [file pone.0248484.s008.doc]

December 9, 2020

Reviewers

*Plos One*

Dear editor and reviewers:

Thank you for the opportunity to review our manuscript. We appreciate your accurate comments, which provided useful insights to improve our review. We present our responses in bold after every comment and highlight the changes in the manuscript in blue. We consider that we have further enhanced the clarity and lightness of our manuscript. We hope that this revised version reflects all the comments and is better suited to your journal for publication.

**Reviewer #1:** Thank you for the invitation to review this paper. Our author colleagues have submitted an interesting systematic review with meta-analysis on the efficacy of electrophysical modalities in the rehabilitation of radial, ulnar, and median neuropathies. The paper concludes that there is limited favourable evidence for efficacy of electrophysical modalities in these conditions.

The authors should be commended for the comprehensive and detailed review they have undertaken.

**We are grateful for the reviewer's insightful comments. We made every effort to address the reviewer's suggestions throughout the manuscript.**

I have some comments for consideration:

Introduction:

1. Opening sentence: ‘Peripheral neuropathies are common injuries’ I suggest use pathologies rather than injury, in case of insidious onset peripheral neuropathies not attributable to trauma.

**We replace the term ‘lesions’ with ‘pathologies’ in the Introduction’s opening sentence, on page 2, line 49**.

2. Paragraph 2, sentences 1 and 2: Citations should be provided for these statements ‘Current literature has focused on the efficacy of surgical and pharmacological treatments. Regarding conservative treatments, most studies evaluated the effects of electrophysical modalities in carpal tunnel syndrome.’

**We provided the missing citations for the statements on page 3, lines 62 and 63**.

Methods:

3. Search strategy – Search was undertaken in April-Jul 2019. This was a while ago, re-running and updating the search should be considered.

**We reactivate the search on 1/10/2020 and adjust the margin until 31/12/2020.**

4. Search strategy – what is the rationale for using just 2 databases? Also, what was the rationale for limiting it to 2009?

**Initially, we chose to focus our review on the last decade’s findings in the two databases with the largest number of clinical trials. However, in order to have a broader perspective, we expanded the search to the following databases: Biomed Central, Ebscohost, Lilacs, Ovid, Pedro, Sage, Scopus, Science Direct, Semantic Scholar, Taylor & Francis, and Web of Science. We also adjust the period of publication from 1980 to 2020. The search strategy is on page 4. We provide an example of the search terms in S2 File.**

5. Study selection and data extraction – indicate which authors (by initials) did the screening, and same for data extraction. Were disagreements solved by consensus discussion?

**As suggested, we include the initials of the authors who did the process. Discrepancies were resolved through discussion and consensus moderated by a third author. Data section, pages 4 and 5.**

6. Study selection and data extraction – were corresponding authors of papers contacted if data were missing or presented in graphs only?

**We contacted the authors but did not respond**.

7. Sections of the Method that are included in the Prisma guidelines are missing – the details of the methods used for summary measures, synthesis of results (e.g. meta-analysis, best evidence synthesis) and risk of bias across studies are not described. Looking at the results, meta-analysis to calculate SMD has been performed, and there is mention of limited and moderate evidence. But these have not been defined or described in the method.

**We modified the Methods section following the PRISMA methodology checklist, pages 3, 4, and 5. We also included the information of the heterogeneity analysis in Data synthesis, page 5.**

8. In line with comment 7 above, meta-analysis was undertaken but there does not appear to be an assessment of the risk of bias across studies or overall confidence of the evidence (e.g. the GRADE approach). This should be done for each meta-analysis comparison to provide insight on the quality of meta-analysis evidence and confidence in the findings.

**We assessed the risk of bias using the Cochrane Risk of Bias Tool, as described on page 5 and S3 Table.**

Results:

9. Paragraph 1 – Science direct and research gate are mentioned here, but were not mentioned in the Method. Were these used as additional databases for the search?

**We obtained the articles from Science Direct and Research Gate from the references of some studies included in the previous review. However, based on the suggestion of expanding our sources, we have included the Science Direct database in this second review.**

10. Reporting of the Results would be much clearer if it were arranged using the sections defined in the Prisma checklist. For example, the section labelled Evidence Hierarchy appears to detail the Study Characteristics. Risk of Bias of individual studies is currently reported in the Method section, so should be presented in the Results instead. Splitting the reporting of results into sections detailing results of individual studies, then detailing the synthesis of results (i.e. meta-analysis and qualitative synthesis) in accordance with the Prisma guidelines would be a much neater way for arranging the Results.

**Thanks for the feedback. As suggested, we report our results using the sections of the PRISMA checklist. We present the findings for each type of comparison and then the meta-analysis of each one. Pages 5 to 24.**

11. The paper is largely well-written, however there are a few grammatical errors (e.g. LLLT is defined twice on page 6), so I suggest reviewing the manuscript for such issues.

**We followed the grammatical suggestions and named the modalities with acronyms only at the beginning.**

12. Forest plots – suggest adding labels to the X axis to show which direction favours the treatment and which direction favours controls

**We add the label on the x-axis of each forest plot indicating the direction of favourability of the treatments.**

13. Meta-analysis text results – suggest including I-squared when reporting the SMD meta-analysis results, to show heterogeneity without needing to refer to the supplementary material.

**We included in each forest plot the heterogeneity results of the I^2^ tests.**

Discussion:

14. The discussion is largely a summary of the results. It would be strengthen the paper to discuss how these findings relate to other studies, consider clinical implications etc.

**We wrote the discussion in terms of clinical relevance contrasting our findings with recent reviews on the topic.**

**Reviewer #2:**

The authors provide a systematic analyses of electrophysical therapy in peripheral nerve entrapment injuries. They included all papers from the course of the last decade and conclude a lack of evidence about the efficacy of electrophysical therapy.

Prisma guidelines were used to prepare the manuscript. I think it is an interesting topic to evaluate non-surgical treatment of peripheral nerve entrapments. Unfortunately the manuscript is very exhausting to read. The authors sum up every included study with a lack of detailed discussion (for example is splinting a viable option, etc.) Preclinical data about the efficacy is not discussed and compared to clinical experience.

There is a lot of information included in an enumerated fashion.

I would suggest to rewrite the manuscript to catch the reader’s attention more and meet the high standard of the journal

**We appreciate your valuable feedback, which allowed us to readjust our review. We describe the results according to the comparison types, clearly stating the findings obtained for all the parameters evaluated and their respective meta-analyses.**

**We rewrote the discussion contrasting our findings with those obtained in the latest reviews of the topic. We compare all the results with the references of the minimal clinically important differences of each of the evaluated parameters. We rewrote a large part of the manuscript considering the suggestions to catch the reader’s attention and meet the high standard of the journal. We hope that we have succeeded.**

Sincerely,

Ena Lucía Bula Oyola

Universidad del Norte

Universitat Politècnica de València

+34 667045622

oyolae@uninorte.edu.co
