# Supplementary material for: Effectiveness of electrophysical modalities in the sensorimotor rehabilitation of radial, ulnar, and median neuropathies: A meta-analysis
Source: PLoS One. 2021 Mar 18;16(3):e0248484. doi: 10.1371/journal.pone.0248484 (PMC7971482; doi:10.1371/journal.pone.0248484)
Supplement: Attachment — Submitted filename: Response to Reviewers.doc [file pone.0248484.s009.doc]

February 13, 2021

Reviewers

*Plos One*

Dear editor and reviewers:

Thank you for this new opportunity to review our manuscript. We appreciate your thoughtful comments which have contributed to the substantial improvement of our revision. We present our responses in bold after each comment and highlight changes to the manuscript in blue. We believe that we have improved the methods of evidence assessment and the discussion of results. We hope that this revised version reflects all comments and is better suited to your journal for publication.

**Reviewer #1:** Thank you for the invitation to re-review this manuscript.

I wish to thank the authors for their time and efforts revising the manuscript, I agree that it is substantially improved in its revised form. Overall, this is a thorough and comprehensive piece of work that will make a valuable contribution to the field.

**We are grateful for the reviewer's insightful comments. We made every effort to address the reviewer's suggestions throughout the manuscript.**

Please see below some comments for consideration:

1) Abstract: ‘We found limited evidence favouring therapies with extracorporeal shock-wave, pulsed radiofrequency, and low-level laser.’ It isn’t clear if this refers to limited quality of evidence, or limited effect size. Please revise to make this more apparent.

**As suggested, we describe our results based on effect sizes and MCID.**

2) Abstract: missing full stop from final sentence of conclusion

**We have included the missing period at the end of the sentence.**

3) Introduction: ‘Symptoms may include partial or total motor dysfunction of the forearm and hand, loss of muscle tone and strength, hypoesthesia or hyperesthesia, pain, allodynia, or paraesthesia’ A minor point, these are both signs and symptoms, so please start this sentence with ‘Signs and symptoms may include…’

**We start the paragraph with the phrase ‘Signs and symptoms may include…’**

4) Methods: For clarity in the Data Synthesis section, please state explicitly that this refers to the meta-analysis performed. Also, some comment on interpretation of effect sizes (small, large etc) would be good, and this would help with the clinical interpretation of findings, and could be integrated/mentioned through the reporting of results (or at least in the Discussion).

**We rewrite the data synthesis section detailing the methods for estimating the effect sizes of the meta-analysis results. This information was also included in the results and discussion.**

5) Methods: The authors have used Cochrane Risk of Bias Tool for assessment of Risk of bias in individual studies (nicely shown in Table S3), however there is no assessment made of risk of bias across studies (like the GRADE approach) to consider confidence in the findings of the meta-analysis comparisons. This would strengthen the review and the manuscript if possible to include.

**We assess the quality of the evidence through the GRADE approach. This information was included in the results, the discussion and the summary of the findings are present as supplementary material in Table S3.**

6) Results: Table S4 – the authors should be commended on the time and work required to produce this comprehensive summary of the results of all included findings. A minor note, it is a bit confusing having Appendix 1 written inside Table S4, suggest just using the term Table S4 rather than Appendix 1

**We replace the term appendix by the name of the table. (Now is named as Table S5).**

7) Results: Paragraph 1 in subheading ‘Effects of electrophysical interventions’ – Is there a parenthesis missing somewhere?

**We have included the missing parentheses. Thank you for your willingness to improve the details.**

8) Results: Forest plots – there are instances of a paper appearing twice within the same forest plot. Why is this? It should be made clearer why this is the case in the caption of each figure.

**We have included the clarifying information as a caption on the image. We list studies that compared more than one treatment modality or dose.**

9) Results: I like the comparison of the meta-analysis findings with MCID, that is a nice way to help convey the clinical significance of the findings. Was this done using the mean differences for the outcomes (rather than SMDs) calculated in the meta-analysis? If so, it would be helpful for these data to be included (even just reported as a range in the text) for the outcomes mentioned in the Clinical Significance paragraph (p25, paragraph 1).

**We describe the information about the MCID estimate and include a reference table with the values.**

10) Discussion: the Discussion succinctly compares findings of the present review and considers the clinical relevance of findings, with respect to the MCID as per comment 9. As noted in comment 4 above, discussing the effect sizes reflected by the SMDs would be another way to explore the clinical significance of these results (e.g. small effect, large effect etc).

**We reinforced the discussion about the clinical significance of the results by contrasting them with effect sizes and MCID**

11) Discussion: How much confidence do the authors have in the results of their meta-analysis comparisons? The discussion of the results largely indicate that electrophysical modalities do not produce clinically significant treatment effects. Considering the quality of the studies that comprise the meta-analysis (plus numbers of participants, effect sizes etc), is this ‘good quality’ meta-analysis data? The GRADE approach referred to in comment 5 is a nice way to consider this should the authors elect to utilise it.

**We include the assessment of the quality of the evidence as an opening for the discussion and highlighting its implications in the effect estimation.**

12) Figures: there are a lot of figures, but they are well done and clear.

**We appreciate your feedback.**

**Reviewer #2:** The authors made a substantial effort to improve their manuscript. The data presentation and discussion gained readability.

**Thanks for your valuable comments.**

I found some minor potential for improvement:

Line 165n and ongoing paragraph should be written in complete sentences and not only in a abbreviated manner.

**We replace abbreviations with full names of treatments and neuropathies.**

Figure 9: All other figures show the sham procedure on the right side of the figure this one on the left. This might be confusing for the Reader. Figure 12: same

**As suggested, we modify the forest plots (Figure 9 and 12).**

Sincerely,

Ena Lucía Bula Oyola

Universidad del Norte

Universitat Politècnica de València

+34 667045622

oyolae@uninorte.edu.co
